# Does structural form matter? A comparative analysis of pooled procurement mechanisms for health commodities

**DOI:** 10.1186/s12992-023-00974-1

**Published:** 2023-11-23

**Authors:** Koray Parmaksiz, Hester van de Bovenkamp, Roland Bal

**Affiliations:** Erasmus School of Health Policy & Management, Erasmus University Rotterdam, Rotterdam, The Netherlands

**Keywords:** Pooled procurement, Joint procurement, Centralized procurement, Organisation of the eastern caribbean states (OECS), Pacific island countries (PIC), Asthma drug facility (ADF), Global drug facility (GDF), Developmental stages, Pharmaceuticals, Medicines

## Abstract

**Introduction:**

Pooled procurement can be seen as a collaboration initiative of buyers. Such mechanisms have received increased attention during the Covid-19 pandemic to improve access to affordable and quality-assured health commodities. The structural form of pooled procurement mechanisms ranges from a third-party organization that procures on behalf of its buyers to a buyer’s owned mechanism in which buyers operate more collaboratively. However, little is known about how these types of pooled procurement mechanisms differ in terms of characteristics, implementation and developmental process. To fill this gap, we compared four pooled procurement mechanisms. Two buyer’s owned mechanisms: the Organisation of the Eastern Caribbean States (OECS) and the Pacific Island Countries (PIC). And two third-party mechanisms: the Global Drug Facility (GDF) and the Asthma Drug Facility (ADF).

**Methods:**

For this qualitative study, we used a multiple case-study design. The cases were purposefully selected, based on a most-similar case study design. We used the Pooled Procurement Guidance to collect data on individual cases and compared our findings between the case studies. For our analysis, we drew upon peer-reviewed academic articles, grey literature documents and 9 semi-structured interviews with procurement experts.

**Results:**

Buyers within a buyer’s owned mechanisms differ in procurement systems, financing structures, product needs and regulatory and legal frameworks. Therefore, buyers within such mechanisms require relative alignment on motivations, goals and operations of the mechanism. Our study showed that buyers’ relative homogeneity of characteristics and their perceived urgency of the problems was particularly relevant for achieving that alignment.

Third-party organization mechanisms require less alignment and consensus-building between buyers. To participate, buyers need to align with the operations of the third-party organization, instead of other buyers. Elements that were essential for the successful implementation and operation of such mechanisms included the procurement secretariat’s ability to create local and global awareness around the problem, to induce political will to act upon the problem, to mobilize sufficient funding and to attract qualified staff.

**Conclusion:**

To successfully sustain pooled procurement mechanisms over time, key actors should drive the mechanism through continuous and reflexive work on stakeholder engagement, mobilization of funding and alignment of interests and needs.

**Supplementary Information:**

The online version contains supplementary material available at 10.1186/s12992-023-00974-1.

## Introduction

The Covid-19 pandemic exposed the fragility of global supply chains of medicine and medical products. Scarcity of raw materials, lockdowns, export bans and shortages of finished products such as vaccines and personal protective equipment caused a ripple effect of supply chain disruptions, which ultimately led to shortages, price increases, quality issues and waste of resources [1–5]. To minimize the effect of these supply-side issues, pooled procurement mechanisms—collaboration initiatives of at least two buyers or a third-party organization that procures on behalf of its participating members [6]—regained global attention. As a result, buyer-countries bundled their financial and technical resources to increase their market size and financial capacity. Examples of such mechanisms during the Covid-19 pandemic include the European Union (EU) Joint Procurement Agreement (JPA) [7], the African Union’s (AU) Africa Medical Supplies Platform (AMSP) [8], and the Covax initiative’s pooled procurement of Covid-19 vaccines [9]. Aided by the nature of the crisis, buyers managed to set up these pooled procurement mechanisms in an exceptionally short time frame.

Pooled procurement during the Covid-19 pandemic was helped by the shared sense of urgency as well as the need for a single health product. Such conditions are often lacking during non-pandemic times, delaying or even preventing such mechanisms to come to fruition. Pooled procurement mechanisms are often seen as a uniform approach to increase access to affordable and quality-assured health commodities. However, a recent review [6] showed that pooled procurement mechanisms are complex, multi-component and context specific mechanisms that vary in structural form (from buyer’s owned/inter-buyer to third-party), operational level (i.e., sub-national, national, inter-country and global), and product types (e.g., patented products, disease-specific products, vaccines or a wide range of essential medicines). Pooled procurement mechanisms also evolve over time. Parmaksiz et al. [10] identified four general developmental stages: the promise stage, the creation stage, the early operational stage and the mature stage. With each stage, the main goal evolves from reaching a shared vision, to developing a shared plan, to establishing a shared practice and finally reaching a sustainable practice. To evolve from one stage to another, continuous effort is required from participating actors to maintain alignment on the various motivations, goals and operations.

Certain types of pooled procurement mechanisms seem to be more complex to set up and sustain compared to others. For example, several successful examples of disease- or product-specific, third-party pooled procurement mechanisms, in which the procurement organization procures on behalf of its buyers, exist. These include the Global Fund (GF) and the Global Alliance for Vaccines and Immunizations (GAVI). Inter-country, buyer’s owned mechanisms that aim to procure a wide range of essential medicines, however, are more complex to set up because countries with often diverging procurement and financing structures, product needs and legal frameworks are more likely to complicate the process of aligning interests, needs and operations [6, 10].

Following Parmaksiz et al. [10], we noticed a lack of academic publications that explore and compare the creation and operational processes within and between different structural forms. Although pooled procurement mechanisms are being promoted as a solution to reduce prices, increase availability, and achieve more efficient procurement processes, there is still limited knowledge on how they function and what makes them successful or not. This paper contributes to filling that gap by increasing our understanding of creating and maintaining pooled procurement mechanisms of health commodities through comparing successful and failed examples of both inter-buyer and third-party pooled procurement mechanisms by answering the following research questions:

1. What elements help to understand successful implementation and operation of both inter-buyer and third-party pooled procurement mechanisms?
2. How do inter-buyer and third-party pooled procurement mechanisms differ in terms of characteristics and developmental process?

To answer these questions, we conducted a comparative case study of four cases. These cases include two buyer’s owned mechanisms, of which one is still active, the Organisation of the Eastern Caribbean States (OECS), and one inactive, the Pacific Island Countries (PIC). And two third-party pooled procurement mechanisms, of which one is still active, the Global Drug Facility (GDF), and one inactive, the Asthma Drug Facility (ADF).

In the following methodology section, we expand on the multiple case-study design that we have used. Then, we present our results, in which we compare the four pooled procurement mechanisms and explore which critical factors played a role in their success or failure. Finally, we summarize our main findings and provide a reflection in the discussion section.

## Methods

### Study design

We used a multiple case-study design. This study design allowed for in-depth analysis of individual cases, while providing an opportunity to compare the effects of contextual factors on the implementation and operations of purposefully selected pooled procurement mechanisms [11, 12].

The cases were purposefully selected, based on a most-similar case study design. In this approach, cases for comparison are selected based on many similar characteristics they share. The relevant characteristics for our study include geography, economic level, population size, type of products they aim to procure and structural form of the pooled procurement mechanism. The most-similar case study design allows researchers to focus on factors that differ between cases that might explain the contrast in their outcome variable, which in this case was the status of the pooled procurement mechanism: active or inactive [12, 13]. Another important selection criterion was the availability of sufficient data in the public domain such as websites, meeting reports, feasibility studies, presentations and peer-reviewed academic papers.

### Case selection

For our study, we compared four pooled procurement mechanisms. Table 1 provides general characteristics of the pooled procurement mechanisms that have been included in our analysis. We selected and paired two buyer’s-owned inter-country pooled procurement mechanisms (The Organisation of the Eastern Caribbean States and the Pacific Island Countries). Comparing these cases was interesting because both pooled procurement mechanisms consist of island nations that share many similarities in terms of population and market size, geographical remoteness, type of products to be pooled and financial capacity, but experienced different life cycles.

We also selected and paired two third-party global health organisation pooled procurement mechanisms (the Global Drug Facility and the Asthma Drug Facility). Our main reason to compare these third-party mechanisms was because the ADF was set up based on the initial success of the GDF, but again, with a different outcome.

The included mechanisms operate or have stopped operating during different developmental stages (cf. [10]). The PIC and ADF have stopped existing in the creation and early operational stage, respectively. The two mechanisms included in our study that are currently still active (i.e., OECS and GDF) both operate in the mature stage.

**Table 1:** General characteristics of the pooled procurement mechanisms included in this study. The characteristics in brackets represent intentions during initial discussions because the PIC pooled procurement mechanism never came to fruition

Characteristics	OECS	PIC	GDF	ADF
**Status**	Active	Inactive	Active	Inactive
**Developmental stage**	Mature	Creation	Mature	Early operational
**Geographical level**	Inter-country	Inter-country	Global	Global
**Type of products**	Essential medicines [multi-product]	(Essential medicines) [multi-product]	TB treatment & diagnostics [disease specific]	Asthma treatment & diagnostics [disease specific]
**Level of collaboration**	Central contracting	(Group contracting)	Central contracting	Central contracting
**Structural form**	Buyer’s	(Buyer’s)	Third-party	Third-party

### Data collection

To assist our data collection and analysis, we used the Pooled Procurement Guidance developed by Parmaksiz et al. [10]. This Guidance document consists of two parts. Part 1 identifies essential elements of pooled procurement mechanisms organized around buyers, pooled procurement secretariat and suppliers. Part 2 provides an overview of the general development of pooled procurement mechanisms over time. The Pooled Procurement Guidance has been developed based on theoretical insights from organizational life cycle literature and collaborative and network governance, semi-structured interviews with procurement experts, and academic and grey literature documents on pooled procurement of medicines and vaccines [10].

First, we collected data on the historical development and the implementation of each pooled procurement mechanism. For the purpose of our study, we mainly focused on the elements identified under Part 1 of the Guidance. For the inter-buyer mechanisms (i.e., OECS and PIC), we looked at all identified elements in this part of the Guidance. For the third-party pooled procurement mechanisms (i.e., GDF and ADF), we tried to collect data on all but elements A6-A17. These elements are mainly relevant to share among buyers in an inter-buyer mechanism, and therefore do not apply to buyers in a third-party, global health organization mechanism.

Data was collected from various sources to triangulate our findings, including grey literature documents, academic literature and 9 semi-structured interviews. We scanned grey literature documents in the public domain about these mechanisms such as annual reports, feasibility studies, evaluation and review reports, news articles and presentations. Databases used to collect these data included official websites and data repositories of the included pooled procurement mechanism, Google Scholar, PubMed, WHO’s Institutional Repository for Information Sharing (IRIS) and news outlets. In addition, we used snowballing by scanning references of both academic and grey literature documents to search for relevant sources. Search terms we used included variations of: pooled, joint, group, centralized, bulk, procurement, and purchasing in combination with medicines and pharmaceuticals. We used Boolean operators to combine these search terms in the databases. Data collection took place between February 2021 and March 2023.

The semi-structured interviews, which were carried out as part of an earlier comparative case study [14] between April and May 2020, were held with purposefully selected respondents with expertise on the implementation and operations of the Global Drug Facility (GDF) and Asthma Drug Facility (ADF). During this study [14], 9 respondents were asked to provide insight on the emergence, development and operations of these mechanisms. 1 procurement specialist/consultant, 1 academic and 7 respondents who worked as a procurement agent were interviewed. The interviews were held online and conducted in English, lasting between 60 and 120 minutes. Written informed consent was obtained for interviews and permission was requested for audio recording. The respondents were pseudonymised using identification numbers and these numbers were stored separately from the transcripts.

Unfortunately, we did not manage to carry out additional interviews for the other case studies included (i.e., OECS and PIC) because we did not get a reply from potential key respondents to participate in semi-structured interviews and we experienced difficulty in reaching out to the respondents that were involved during the time of preliminary discussions, especially for the Pacific Island Countries. However, the volume of data and triangulation of data sources increases our confidence in the validity and reliability of our study data.

### Data analysis

We analysed the data using mainly a deductive approach, which allowed for testing the earlier developed Pooled Procurement Guidance in practice and explaining why certain elements were more relevant for the emergence, development and the success or failure of the included pooled procurement mechanisms. We used a thematic synthesis approach for the cross-case comparison. This approach allowed us to identify most important themes emerging from the collected data [15].

The structure of Part 1 of the Pooled Procurement Guidance served as the foundation for our data collection and analysis. The elements identified in Part 1 of the Pooled Procurement Guidance were selected as descriptive themes. The collected data in the form of grey literature documents, academic publications and semi-structured interviews on the historical development and the relevant elements identified in the Pooled Procurement Guidance were analysed, summarized and consolidated in a Guidance table. These in-depth case-studies and reference lists of each selected pooled procurement mechanism have been added as supplementary materials (Additional files 1, 2, 3 and 4).

After the in-depth individual case studies, we compared our findings within each pair of pooled procurement mechanism (i.e., ADF vs. GDF and OECS vs. PIC). This allowed us to explore why certain elements appeared to be more essential in the emergence, development and operations of certain pooled procurement mechanisms compared to others. After that, we also compared between the case-study pairs (i.e., ADF/GDF vs. OECS/PIC), to explore if there were any elements that were particularly essential for a certain structural form of pooled procurement mechanism (buyer’s owned vs. third-party).

We then held multiple sessions within the research team until consensus was reached on the relevant analytic themes. The analytic themes that emerged from the thematic analysis included: (1) the problem, the politics and the motivations to participate; (2) Securing sufficient, predictable and timely budget; (3) Continuous alignment of goals, purpose and operations; (4) Clear organizational structure of the secretariat; and (5) Incentives for suppliers. NVivo (12.7.0) was used as the qualitative data analysis software.

## Results

Our analysis is based on case studies of four pooled procurement mechanisms: The Organisation of the Eastern Caribbean States (OECS), the Pacific Island Countries (PIC), the Global Drug Facility (GDF) and the Asthma Drug Facility (ADF). Further information on general characteristics, historical background and in-depth analyses of each individual pooled procurement mechanism can be found in the supplementary materials (Additional files 1, 2, 3 and 4).

 In the following sections, we have highlighted important findings that emerged from our comparative case-study analysis, explaining the success or failure of the included pooled procurement mechanisms. We explore how the key components of a pooled procurement mechanism, which include the way the buyers, the budget, the pooled procurement organization and suppliers are organized, incentivized and interact with each other. In this section, we do not expand on all essential elements identified in the Pooled Procurement Guidance [10] for each case-study. Instead, we highlight the essential elements that were critical for the success or failure of these mechanisms. A comprehensive overview of the presence, partial presence or absence of the essential elements for each case study can be found in Table 2 and in the supplementary materials.

**Table 2:** Comparative overview of the presence, partial presence and absence of ﻿the essential elements within the case studies

Essential elements/actor	OECS	PIC	GDF	ADF
*Status*	*Active*	*Inactive*	*Active*	*Inactive*
A. **Buyers**				
** All buyers need to have individually:**				
1. Perceived problem for which pooled procurement may be a solution (problem)				
2. Motivations that outweigh the opportunity costs				
3. Budget, either internal or external (through donors)				
4. Sufficient technical capacity (e.g., demand forecasting)				
5. Compatible laws, regulations and policies that allow for (international) pooled procurement				
** If buyer’s mechanism, all buyers combined, need to have:**				
6. Demonstrated willingness to solve their problem collectively through pooled procurement (shared vision)			**–**	**–**
7. Alignment on goals, purpose and operations of the pooled procurement mechanism (shared plan)			**–**	**–**
8. Joint need for specific products (product alignment)			**–**	**–**
9. Sufficient market			**–**	**–**
10. Sufficient and stable financial capacity (financial capacity)			**–**	**–**
11. Regulatory harmonization (e.g., shared quality standards, joint assessment, mutual recognition, etc.)			**–**	**–**
12. Trust (in other buyers and the pooled procurement organization)			**–**	**–**
13. Transparent data and information sharing			**–**	**–**
14. No history of conflict or failed collaboration			**–**	**–**
15. Homogeneity of buyer’s characteristics related to their needs			**–**	**–**
16. Shared cultural factors and values (e.g., language, traditions, etc.)			**–**	**–**
17. Existing political or structural mechanisms			**–**	**–**
B. **Pooled procurement organization**				
1. Organizational and good governance structure with clear roles and responsibilities		**–**		
2. Clear mandate		**–**		
3. Standardized and transparent procedures		**–**		
4. Sufficient, predictable and timely budget, either internal or external (through donors) to carry out pooled procurement		**–**		
5. Sufficient, predictable and timely budget, either internal (through service fees) or external (through donors), to cover organizational expenses		**–**		
6. Predictable, timely and efficient payment mechanism		**–**		
7. Human resources (sufficient in numbers and expertise)		**–**		
8. Sufficient technical capacity (e.g., procurement, quality assessment, forecasting, etc.)		**–**		
9. Positive reputation		**–**		
10. No conflict of interest		**–**		
11. “User-friendliness” (both towards buyers and sellers)		**–**		
C. **Suppliers**				
1. Sufficient number of qualified suppliers				
2. Sufficient production incentives	**–**	**–**		
3. Sufficient supply incentives				
4. Sufficient number of distributors with favourable delivery terms				

*OECS* (Organisation of the Eastern Caribbean States), *PIC* (Pacific Island Countries), *GDF* (Global Drug Facility), *ADF* (Asthma Drug Facility).

: Present,  : Partially Present,  : Absent,  : No information, **–** Not relevant

### The problem, the politics and motivations to participate

Before establishing any pooled procurement mechanism, it is imperative that potential buyers, such as high burden countries, and other key actors in the global health arena, such as global health organizations and donors, experience the problems for which pooled procurement may be a solution. If and how these key actors experience the problems that need to be solved, depends on multiple factors.

In the case of Tuberculosis (TB), the combination of three main factors created the preconditions to raise global attention: the nature of the disease, the high disease burden, and the possibility to cure TB with the right treatment. TB is an airborne communicable disease caused by the *Mycobacterium tuberculosis*. The bacteria are transmittable from human-to-human and can lead to death if left untreated [16, 17]. TB has a high global disease burden. The World Health Organization (WHO) estimates that a quarter of the world population has a TB infection, with 30 high burden TB countries, mainly low- and middle-income countries, accounting for 90% of the TB cases [17]. Currently, TB is estimated to cause 1.6 million deaths per year [18]. The development of an effective multi-component treatment strategy, called DOTS (*Directly observed treatment, short-course*), was an important breakthrough for curing people with TB [16]. However, many high burden TB countries experienced challenges in implementing the DOTS strategy. These challenges included lack of financial resources to procure TB medicines, lack of access to high quality TB medicines, inefficient procurement systems, lack of national TB programs, a small market size for TB medicines and diagnostics, and limited human resources to accurately diagnose and treat TB [19–21].

In 1993, TB climbed its way up on the global health agenda after the WHO declared TB a global health emergency, acknowledging that the spread of TB could only be contained if a universal approach would be taken [22]. This global attention brought many key actors together, including global health organizations, high burden TB countries, donors and NGOs. One important initiative that emerged from this multi-actor engagement in the battle against TB was the Stop TB Partnership [19, 23]. This partnership led to the establishment of the Global Drug Facility (GDF). The GDF was set up to solve the problems of lacking high quality and affordable TB medicines, which was seen as an important barrier in the rapid expansion of the DOTS strategy [19, 24, 25].

Increased global awareness, the availability of external funding, which we’ll discuss in the next section, and the multi-stakeholder approach put TB also on the national agenda of potential buyers. These buyers realized that pooled procurement, as organized by the GDF, could provide a solution to the problems they experienced in the fight against TB.

In the case of the Asthma Drug Facility (ADF), these preconditions for global awareness and multi-actor engagement were lacking to a large extent. An estimated 262 million people worldwide are affected by asthma causing approximately 461,000 deaths annually [26]. In contrast to TB, however, asthma is a non-communicable disease caused by a chronic inflammation of the airways with no cure. Asthma treatment is mainly focused on symptom relief [27]. The lack of perceived urgency to treat asthma in the global health arena and in potential buyer countries, mainly low- and middle-income countries, have been attributed to multiple factors. In many of the potential buyer countries these include insufficient health services around chronic diseases, poor access to accurate diagnostics, inaccurate demand forecasting of asthma treatments, a lack of context-specific national programs, guidelines and training on asthma, and inadequate knowledge among clinicians and the general public on the diagnosis, disease, treatment and management of asthma [14, 27–33]. One of our respondents confirmed that low- and middle-income countries did not perceive asthma as an urgent problem:

“Countries did not see what problems Asthma Drug Facility was able to solve for them, because they did not see any problem: for them asthma was not an issue.” [Procurement agent].

As a result, the ADF experienced difficulties with creating sufficient global awareness on the problems around treating asthma, constructing a global health infrastructure to congregate key actors, attracting external funding, and convincing potential buyers to focus on detecting and treating asthma. The ADF tried to provide a solution with high quality and affordable medicines to problems that were not equally perceived as urgent by other key actors.

### Motivations to participate

However, even if a pooled procurement mechanism manages to provide a solution to urgent problems, the perceived urgency of the problem in itself is not sufficient for buyers to participate in a pooled procurement mechanism. The motivations to participate in such a mechanism need to outweigh the costs of participation. For example, some Pacific Island Countries (PIC), who procure their medicine from other countries in the Pacific region with whom they have had a strong historical relationship, such as Australia and New Zealand, showed little motivation to participate in an inter-country pooled procurement mechanism [34–36]. In addition, some PIC were concerned that the additional distribution costs of a potential inter-country pooled procurement mechanism, and the high costs of integrating administrative, political and bureaucratic structures would outweigh the financial benefits that the inter-country pooled procurement mechanism would generate [37, 38].

The GDF, on the other hand, does not merely operate as an intermediate organization that consolidates demand and carries out pooled procurement. It also provides a rounded procurement service to incentivize buyers to participate in the mechanism. For example, the GDF provided grants to eligible buyer countries at initiation [19]. Currently, a Flexible Procurement Fund (FPF) provides financial flexibility to buyers that have difficulty to adhere to GDF’s prepayment conditions [39]. The GDF also supports buyer countries with capacity building and technical assistance in several areas, including demand planning and stock monitoring [20, 40, 41]. The GDF and the Stop TB Partnership, under which the GDF is housed, both operate under the United Nations umbrella. This institutional backing legitimized GDF’s operations and provided the GDF high-level access to high burden TB countries. Reciprocally, the GDF involves representatives of high burden TB countries in its governance mechanism as board members of the Stop TB Partnership [23, 42]. This governance structure increased the buyer’s trust in the GDF. GDF’s user-friendly services with high client satisfaction ratings [21], combined with a positive track record of increasing access to quality-assured and affordable TB medicines have reinforced the GDF’s positive reputation to attract and incentivize potential buyers to procure through its pooled procurement organization.

The findings show us that in order to establish pooled procurement mechanisms successfully, it is essential for buyers and other key actors in the global health arena to experience an urgent problem for which pooled procurement may provide a solution. This perceived problem should generate sufficient political will from buyers. In addition, the buyers’ motivations to participate in the mechanism should outweigh their costs of participation. Finally, the proposed solution (i.e., the operational model of the pooled procurement mechanism) should be compatible with the experienced problems.

### Securing sufficient, predictable and timely budget

The presence of sufficient, predictable and timely budget, either internal or external (i.e., through donors) is needed to both procure medicines and to cover organizational expenses. Securing this budget depends heavily on the key actors’ perception of the problem, their motivations to participate and their political willingness and power to act on the problem, as discussed in the previous section. In addition, this budget is required at all levels (i.e., the buyer level, the inter-country level, and the pooled procurement organization level), which we will further specify in the following section.

Allocating internal budget at the buyer’s level relies on the potential buyer’s perceived urgency of the problems that the pooled procurement mechanism aims to solve. Often, the limited internal budget that is available in low- and middle-income countries, especially for non-communicable diseases, has to compete with other more urgently perceived (communicable) diseases, as seen in the examples of the ADF. Attracting external funding relies mainly on the perceived urgency of the problems by donor countries or organization, as seen in the example of the GDF. The nature of TB, which spreads from human-to-human through air poses a potential health threat for higher income countries. This threat, in combination with the high disease burden and the fact that TB is curable with the right treatment, allowed the “TB-sector” to attract a significant amount of external funding from donor organizations and development aid from high-income country foreign ministries.

In buyer’s mechanisms, a collective sufficient and stable budget at the inter-country level is required. The importance of this collective budget became evident in the PIC example, where a lack of agreement and coordination of PIC’s budget was influenced by a lack of agreement on which currency to use and the absence of an appropriate and trusted financial organization in the region that could coordinate the financial process [37, 43].

In contrast, the member countries of the Organisation of the Eastern Caribbean States (OECS) share a common currency and succeeded in establishing a revolving fund at the inter-country level, managed by the Eastern Caribbean Central Bank (ECCB). These preconditions created a trusted and user-friendly mechanism to transfer money resulting in OECS members to deposit one-third of their annual internal pharmaceutical budget to individual country drug accounts at the ECCB [44]. This was a clear demonstration of political will from OECS members at initiation of the mechanism.

The pooled procurement organization or secretariat that carries out the procurement also requires sufficient, predictable and timely budget to both procure medicines and to cover organizational expenses. These organizational expenses might include salaries of staff, office rent, maintaining IT systems, daily management tasks and organizing meetings. While inter-country pooled procurement mechanism like OECS and PIC relied mainly on the buyer’s internal budget, disease-specific third-party pooled procurement organizations were more likely to target external funding to procure medicines. Some were successful like the GDF, and some unsuccessful like the ADF, for the reasons explained above.

Similarly, organizational expenses have been covered with internal budgets, mainly through service fees, or external, through donor funding. The OECS inter-country pooled procurement mechanism was set up in 1986 with initial support from USAID, which provided technical assistance and covered its operating costs for the first couple of years [45, 46]. After the initial funding, the organization became self-sustaining in 1989 through relying on service fees, which was incrementally reduced from 15% to 1986 to its current level of 9% since 2016 [46, 47]. Similarly, the ADF aimed to sustain its secretariat by adding a mark-up to every order [36]. But due to a lack of incoming orders, the International Union Against Tuberculosis and Lung Disease (The Union), under which ADF was housed, had to bear the costs of ADF’s operations. This financial burden eventually resulted in The Union terminating ADF’s operations in 2013, before the ADF could reach its aim of financial sustainability [14]. The GDF, on the other hand, relies mostly on external funding from USAID, secured through congressional budget, to cover organizational expenses of its secretariat [48, 49].

We can conclude from the above that sufficient, predictable and timely budget is needed at all levels of the pooled procurement mechanism, both to procure health products and to cover organizational expenses. Securing sufficient budget is highly connected to the key actors’ perceptions of the problem, their motivations to participate and their political willingness and power to put the problem on the national and global agenda.

### Continuous alignment of goals, purpose and operations

In a buyer’s owned pooled procurement mechanism, in which the buyers manage the operations of the mechanism more collaboratively compared to a third-party organization pooled procurement mechanism, the buyers need to continuously align on goals, purpose and operations of the mechanism. Alignment does not necessarily mean that all buyers need to have the same goals, purpose and operations for the mechanism. But alignment can still be achieved as long as the goals, purpose and operations of different buyers are not conflicting.

In the case of the OECS, all buyer countries experienced similar problems, such as a small market size, limited availability of health products, relatively high costs of procurement of health products, and a limited efficiency of procurement and supply management [50–52]. These shared problems resulted in converging needs for OECS members, facilitating the alignment on goals, purpose and operations of the pooled procurement mechanism. For example, the similarity of characteristics between OECS members in population size, demographics and financial capacity results in a common need to increase their market size and a joint need for specific products. Agreeing on these products is a deliberate process that requires continuous work and alignment. The products are listed in the *Regional Formulary and Therapeutic Manual* and are reviewed annually by the Technical Advisory Committee [50]. In addition, OECS member’s geographical location as remote island nations and their small population size generates the common need to reduce (distribution) costs and increase availability of health products. The relatively high homogeneity of OECS member characteristics related to their needs allowed the OECS to set up a central contracting mechanism, the most integrated form of pooled procurement.

Similar to OECS, the Pacific Island Countries (PIC) consist of a broad range of remote and dispersed island nations in the Pacific Ocean. Unlike OECS, the characteristics of PIC are more heterogeneous related to their needs. This heterogeneity is particularly apparent when comparing the PIC’s population size, demographics, financial capacity, currency, and health outcomes. which might translate in different needs for health products, increasing the difficulty of product alignment [53–55]. The PIC’s divergence in buyer’s characteristics related to their needs, has contributed to misaligned goals, purpose and motivations among PIC. The motivations for smaller island states (SIS) were mainly to increase access to affordable medicines by increasing their market size, whereas Fiji’s driving force to participate was mainly to become the leading country of the inter-country pooled procurement mechanism, expressing concerns about their sovereignty if they were not provided with this leading role [37]. However, other PIC, such as the Solomon Islands have resisted the idea of Fiji taking a leading role in the inter-country pooled procurement mechanism, partially because of past experiences of failed collaborations, e.g. the attempt in 1971 to set up a joint airline, called Air Pacific which fell apart because many PIC, particularly the Solomon Islands, believed that the airline was mainly benefitting Fiji [56]. Although there have been successful examples of collaboration initiatives in the Pacific region, such as the Forum Fisheries Agency and the South Pacific Tourism Organisation [56], the negative experiences have made PIC reluctant to rely too much on others within the inter-country pooled procurement mechanism. Inevitably, these failed collaborations have impacted the level of trust between Pacific Island Countries. Also, the great diversity in culture, tradition and languages between the PIC might have had an impact on these trust levels [57, 58]. The fragility of the trust level between PIC were underlined recently, when five PIC of the Micronesian sub-group quit the Pacific Islands Forum over a dispute on selecting the Forum’s new Director-General [59]. As seen in the examples above, the history of collaboration among buyers, and the presence of pre-existing organizational and political structures can influence the alignment of buyers and creation of pooled procurement mechanisms. These collaboration efforts can reinforce trust among the buyers, as seen in the OECS example, or reduce trust, as seen in the PIC example.

Our findings show that relative and continuous alignment on products, goals, purpose and operations between buyers is essential. This alignment is heavily influenced by the homogeneity of buyers’ characteristics, their experienced levels of trust and their experiences in collaboration. This is especially important for a buyer’s mechanism, because buyers often participate directly in the management and operations of the pooled procurement mechanism.

### Clear organizational structure of the secretariat

Our analysis showed that the pooled procurement mechanisms that were still operational relied on the presence of a dedicated secretariat with clear roles and responsibilities. For example, the OECS Pharmaceutical Procurement Service (PPS) is run by a permanent secretariat with dedicated staff. All buyer countries are represented through the policy board of the PPS. Procurement is carried out by various committees with clear task divisions and integrated safeguards to limit the possibility of conflicts of interest [44, 45, 50].

Similarly, the GDF is run by a dedicated secretariat with sufficient human resource capacity. The GDF secretariat consist of around 40 staff members, of which around 30 are based at the headquarters in Geneva, Switzerland; and the remaining staff operating externally in different regions, mainly in high burden TB countries. The staff of the GDF are highly trained and highly specialised, focusing on many areas, including TB advocacy, market shaping, sourcing, stakeholder alignment and coordination, demand forecasting and quantification, technical assistance and capacity building, tendering, contract management with suppliers, oversight of quality assurance, warehousing, distribution, and data management [60]. GDF’s organizational structure with a dedicated secretariat, sufficient in numbers and expertise, allows the GDF to provide a rounded procurement service. In addition, the GDF involves representatives of high burden TB countries, global health organizations, international donors and technical agencies in its governance mechanism as members of the Stop TB Partnership Coordinating Board. This structure, where a wide variety of stakeholders take part in GDF’s operations, contributes to legitimizing GDF’s operations in the buyer countries, and provides an arena that facilitates continuous alignment of goals and incentives [23, 61].

In contrast, the Asthma Drug Facility (ADF), another disease-specific third-party organization pooled procurement mechanism, lacked a clear organizational structure and dedicated staff to carry out the pooled procurement of asthma treatment. The ADF was housed under The Union and was run by a small in-house team. However, according to our respondents, ADF’s in-house team had to carry out procurement and related tasks on top of their already existing duties and responsibilities for The Union, impeding their output.

### Incentives for suppliers

Besides buyers, the pooled procurement organization, and a sufficient and predictable source of funding, suppliers are essential for the functioning of the pooled procurement mechanism. The suppliers (i.e., manufacturers, wholesalers and distributors) produce and supply health products to buyers via the pooled procurement organization or secretariat. For suppliers to participate in the mechanism, buyers and the pooled procurement organization need to sufficiently incentivize suppliers. Parmaksiz et al. [10] have made a distinction between production and supply incentives. Production incentives incentivize suppliers to specifically produce products for the buyers in the particular pooled procurement mechanism, whereas supply incentives incentivize suppliers to supply products that are already being produced by the supplier for other markets, to the buyers in the pooled procurement mechanism.

The OECS pooled procurement mechanism was set up to procure relatively high demand essential medicines [44, 50]. Since these products were already being produced in high volumes by suppliers, providing sufficient production incentives was less relevant for OECS members. Instead, OECS members had to provide sufficient supply incentives for suppliers to supply these essential medicines to the Eastern Caribbean Island nations. These supply incentives included a centralized payment mechanism, standardized and transparent procurement processes, enforced participation of OECS members creating a public sector monopsony, a consolidated market, a generally positive reputation, and long-term framework agreements [44, 45, 50, 62].

Although both the OECS and the Pacific Island Countries (PIC) consist of island nations, there are important difference between the two regions. The PIC are more geographically remote and dispersed, increasing delivery times and distribution costs [35, 37]. Also, the prices of medicine in the Pacific Islands region were already relatively low. For example, Vanuatu and Solomon Islands already achieved lowered medicine prices compared to the OECS pooled procurement mechanism [43]. Further, the PIC have a limited financial capacity and lack a unified payment mechanism [37, 43]. In addition, there is no centralized authority or secretariat that facilitates the procurement and/or distribution of health products in the region [43]. Even if the PIC pooled procurement mechanism had managed to evolve from its creation stage into an early operational stage, the PIC would have had to overcome these barriers to provide sufficient supply incentives to potential suppliers.

Providing sufficient production incentives and shaping the market was more relevant for GDF’s pooled procurement mechanism. Prior to GDF, there was a lack of quality generic TB medicines [19, 20]. The GDF has taken multiple market shaping approaches and provided several production incentives to suppliers for the production of quality TB medicines. As part of its market shaping efforts, the GDF accelerated the simplification and standardization of complex TB treatment regimens by consolidating demand in buyer countries around affordable fixed-dose combination (FDC) treatments and incentivizing its production [63]. Similarly, the GDF has played a critical role in driving research and development, procurement and adoption of paediatric TB medicines [21, 64]. The GDF also provided many production incentives to suppliers. For example, the TB medicines market, which was too small and unpredictable, made it risky and expensive for suppliers to carry stock. The GDF tackled this by establishing a Strategic Rotating Stockpile (SRS). The SRS created a buffer stock and levelled off the erratic demand of buyers, resulting in sharing the risk of stock carrying with suppliers and reduced delivery lead times of TB medicines [65]. The GDF also provided suppliers long-term framework agreements giving suppliers a certain degree of security to produce, as long as they adhered to the agreed conditions and quality standards [66]. Also, the quality of mainly domestically produced TB medicines was improved in close collaboration with the WHO Prequalification Programme. The GDF achieved annual fee exemption of TB medicines with relatively low profit margins from the WHO Prequalification program, lowering the barrier for manufacturers to obtain prequalification for their products and making the production of prequalified TB products economically feasible [65]. This fee-exemption and GDF’s quality-assurance policy requirements forced suppliers to adhere to either WHO-prequalification or Stringent Regulatory Authority standards if suppliers wanted to get access to a consolidated TB medicines market provided by the GDF [67].

In addition to production incentives, the GDF also provides suppliers with several supply incentives, such as the adoption of a predictable, timely and single currency payment mechanism to facilitate prompt payment of suppliers and the packaging of TB medicines in 4 languages by the GDF to reduce the supplier’s burden of repackaging and translating [41, 68].

The ADF, on the other hand, struggled with shaping the market for asthma treatments and providing sufficient production incentives to suppliers. The ADF tried to expand the market for asthma inhalers in lower- and middle-income countries and incentivize suppliers to produce and supply quality and affordable asthma medicines to these markets. However, in part due to the complexity of the technology of asthma inhalers, there were insufficient incentives for new generics manufacturers to produce asthma inhalers resulting in a relatively low competition in the market [31, 69, 70]. Although production incentives were limited, ADF’s promise of consolidating previously non-existing markets in lower- and middle-income countries did provide sufficient supply incentives for some manufacturers that were already producing single ingredient pressurized metered dose inhalers. Several of these manufacturers, both originator and generics, entered ADF’s qualification process [69]. According to our respondents, generics manufacturers participated in ADF’s qualification process because they perceived the ADF as an opportunity to enter new markets that were traditionally dominated by originator companies, whereas the originator manufacturer considered the ADF’s mechanism as a form of tiered pricing policy to reach ‘low- and middle-income’ markets in addition to their already secured ‘high-income’ markets.

ADF’s initial efforts resulted in significant price reduction of asthma medicines and inhalers, reaching more than 50% reduction of annual costs per patient with severe asthma in some countries [36, 71, 72]. However, despite ADF’s initial successes in reducing prices and incentivizing manufacturers to supply asthma products, these incentives were not sufficient. The ADF did not reach a sufficient market size to manage a sustainable pooled procurement mechanism, because asthma was not perceived as an urgent problem that needed to be solved by potential buyer countries, resulting in a lack of demand. In addition, potential buyer countries lacked the financial capacity to procure asthma medicines through ADF, while the ADF failed to attract sufficient donor funding due to the nature of the disease.

Our results show us that in order to operate a sustainable pooled procurement mechanism, suppliers need to be incentivized to participate in the pooled procurement mechanism, both in terms of production and supply.

## Discussion

In this article, we compared the implementation and operation of two types of pooled procurement mechanisms: inter-buyer and third-party organization mechanisms. These insights are valuable as inter-buyer mechanisms are often set-up inspired by success stories of third-party pooled procurement mechanisms, but with limited success to date due to the additional complexity that is involved in setting up such inter-buyer mechanisms. In this section, we reflect on why some pooled procurement mechanisms were successful while others were not, with specific attention to how inter-buyer mechanisms differ from third-party organization mechanisms in their characteristics and developmental process.

As we have argued, potential buyers and other key actors in the global health arena should experience or face a problem for which pooled procurement may be a solution. In the absence of such an explicit problem, the mechanism might struggle to get off the ground or endure. As was seen in the example of the ADF, their efforts to create national and global awareness and engage stakeholders was a short-lived success. Putting a problem successfully on the national and/or global agenda is highly political and directly linked with the ability to mobilize sufficient funding. Asthma, which is a non-communicable disease without cure, suffered from low detection rates in potential buyer-countries because of a lack of accurate diagnostics. The ADF struggled to create local political awareness and convince potential buyers to allocate sustainable national budget to asthma treatment. The ADF also failed to attract international donor funding to procure medicines and cover organizational expenses. We argue that this might have been influenced by the complexity of treatment options and lower prioritization of non-communicable diseases over communicable diseases by global health donors [73]. The GDF, on the other hand, had a better recipe for success. High burden of disease in potential buyer-countries combined with accurate diagnostics, effective treatment regimen and potential threat to global health security provided favourable breeding grounds for multi-actor engagement, political determination to act upon the problem, mobilization of funding and attracting sufficient and qualified staff.

This research also shows us that establishing an inter-buyer mechanism, such as the OECS and PIC, is often more complex than third-party organization mechanisms because each participating buyer-country has its own procurement systems, financing structures, product needs and regulatory and legal frameworks that need relative alignment among buyers. Buyers’ relative homogeneity of characteristics and their perceived urgency of the problems was particularly important as it facilitated alignment on motivations, goals and operations of the mechanism between buyers. Success depends on alignment, but not perfect agreement. Buyers can have differences in operations and motivations to participate, as long as these do not clash with the goals and operations of the designated pooled procurement secretariat. The example of the PIC shows that this alignment process can be influenced by various factors. The diverging characteristics of buyers (e.g., population size, demographics, financial capacity) in combination with a history of failed collaboration examples (e.g., Air Pacific) and a fear of loss of sovereignty have reduced trust levels among potential buyers. In turn, the lack of political determination and the non-existence of an entity that takes responsibility to drive the mechanism forward [58], has resulted in the collapse of the negotiations on inter-country pooled procurement between the Pacific Island Countries. In addition, the motivations to participate in a mechanism should outweigh the costs of participation. Participation should generate a net benefit.

Creating a window of opportunity to align interests, however, is not a singular event. The incentives of buyers might change, based on changes in the political, economic, health and demographic environment. In addition, the pharmaceutical market or the international context the mechanism operates in, might change. Preserving alignment is a continuous and reflexive process that requires broad involvement, work and support by the buyers in the mechanism [10]. To facilitate this consensus-building, specific infrastructure such as forums, committees and advisory boards, as seen in the examples of the OECS and GDF, need to be in place.

In contrast to inter-buyer mechanisms, however, third-party organization mechanisms require less involvement of buyers in their operations and therefore less consensus-building among buyers. On the one hand, the governance structure of these mechanisms is often less complex, because each buyer should align with the operations of the pooled procurement secretariat (e.g., ADF, GDF), but not necessarily with other buyers. While on the other hand, setting up such mechanisms requires additional work and effort for third-party organization mechanisms because they are often not initiated by buyer-countries themselves. As we have seen, the ADF was launched by The Union, while the Global Drug Facility was set up with great efforts of the WHO. Because buyers are not directly involved in the creation of third-party pooled procurement mechanisms, the procurement secretariat lacks a guaranteed market to sell their products in the early operational stage. The procurement secretariat’s ability to create a consolidated market by raising local awareness and convince potential buyers to procure through their secretariat is critical for their survival.

In our analysis, we did not address each essential element identified in the Pooled Procurement Guidance, as shown in Table 2, separately. To facilitate our comparative analysis, we focused on elements that played a critical role in the success or failure of one or more mechanisms instead. As a result, some elements might have been under- or overemphasized in explaining the process of setting up and operating each case study individually. We advise readers to interpret our findings with this in mind and consult our supplementary materials (Additional files 1, 2, 3 and 4) for a more complete overview of each essential element’s contribution to the functioning of individual pooled procurement mechanisms.

### Recommendations for applying the pooled procurement guidance

This research shows us that some of the elements identified in the Pooled Procurement Guidance [10] are more political (e.g., urgency of the perceived problem, motivations to participate, creating continuous alignment, incentivizing suppliers), while others are more organizational (e.g., sufficient and predictable budget, sufficient staff and expertise, clear organizational structure). However, these are both strongly related and intertwined in the sense that the political elements influence the organizational elements, and vice versa. Without an urgent problem, there will be no sufficient budget to procure health products and cover organizational expenses, and without a sufficient budget, there will be no suppliers to supply or produce the products.

Further, applying the Pooled Procurement Guidance, as we have done in the supplementary materials (Additional files 1, 2, 3 and 4), is a valuable exercise that can be done during each developmental stage of the pooled procurement mechanism. For those involved in and responsible for setting up pooled procurement mechanisms such as policy makers and consultants, the Pooled Procurement Guidance can provide a structure to align interest and goals. For researchers, the Guidance provides a framework to decompose the complexity around pooled procurement mechanisms. It facilitates identification of elements that contribute to the success or failure of such mechanisms during implementation and operation. Although this exercise provides a comprehensive overview of the elements that might influence the mechanism’s implementation and operations, it remains a snapshot of that particular moment in time of when the exercise was carried out. Therefore, we encourage researchers, policy makers and others to update the document with regular intervals and reflect on the outcomes as the mechanisms evolves over time. We also believe that this Guidance might further benefit from a more detailed, micro-level application on how a single pooled procurement mechanism develops over time. Therefore, we plan further studies to explore how a single pooled procurement mechanism develops over time in order to increase our understanding of why and how specific elements are essential during which developmental stage

### Limitations

Our study might carry a risk of bias. We conducted additional semi-structured interviews with respondents involved in two out of four selected case-studies (i.e., GDF and ADF). This might have resulted in less detailed descriptions and personal experiences on the OECS and PIC case studies. One of our criteria to purposefully select these case studies, other than their similarity in characteristics, was the availability of sufficient data in the public domain. We attempted to overcome this limitation through data triangulation by using a variety of sources including peer-reviewed academic literature and grey literature documents such as official reports, feasibility studies, newspaper articles and presentations as shown in the reference lists of our supplementary files.

## Conclusion

This comparative case-study draws lessons from four purposefully selected pooled procurement mechanisms. We have highlighted why some elements were essential for the implementation and operations of certain pooled procurement mechanisms, while other elements were not. Our findings show that to establish a pooled procurement mechanism, buyers and other key actors should experience an urgent problem for which pooled procurement may provide a solution. This problem should generate sufficient political will from buyers, while the motivations to participate in the mechanism should outweigh their costs of participation. In order to sustain the pooled procurement mechanism successfully, key actors should drive the mechanism through continuous and reflexive work on stakeholder engagement, mobilization of funding and relative alignment on products, goals, purpose and operations, which is influenced by the homogeneity of buyers’ characteristics, their experienced levels of trust and their previous experiences in collaboration.

### Supplementary Information

**Additional file 1.**

**Additional file 2.**

**Additional file 3.**

**Additional file 4.**

## Data Availability

The interview data generated and/or analyzed during the current research are not publicly available as individual privacy could be compromised. All other data is available in the public domain.

## Abbreviations

ADF: Asthma Drug Facility
AU AMSP: African Union Africa Medical Supplies Platform
Covax: COVID-19 Vaccines Global Access
DOTS: Directly observed treatment, short-course
ECCB: Eastern Caribbean Central Bank
EU JPA: European Union Joint Procurement Agreement
FDC: Fixed-dose combination
FPF: Flexible Procurement Fund
GAVI: Global Alliance for Vaccines and Immunizations
GDF: Global Drug Facility
GF: Global Fund to Fight Aids, Tuberculosis and Malaria
IRIS: Institutional Repository for Information Sharing
NGOs: Non-governmental organizations
OECS/PPS: Organisation of the Eastern Caribbean States Pharmaceutical Procurement Service
PIC: Pacific Island Countries
SRS: Strategic Rotating Stockpile
TB: Tuberculosis
The Union: International Union Against Tuberculosis and Lung Disease
USAID: United States Agency for International Development
WHO: World Health Organization
