# Supplementary material for: Does structural form matter? A comparative analysis of pooled procurement mechanisms for health commodities
Source: Global Health. 2023 Nov 23;19:90. doi: 10.1186/s12992-023-00974-1 (PMC10668364; doi:10.1186/s12992-023-00974-1)
Supplement: Supplementary file 1 — Additional file 1. [file 12992_2023_974_MOESM1_ESM.pdf]

Mature stage – Organisation of the Eastern Caribbean States (OECS)

General characteristics

The Organisation of the Eastern Caribbean States (OECS) was founded in 1981 after seven Eastern Caribbean island-nations signed the Treaty of Basseterre. The goal of this treaty was to intensify cooperation and promote unity and solidarity among its members [1]. The Basseterre treaty was revised in 2010 to establish the OECS economic union with a shared currency.

In part due to their remote location and small market size, the Eastern Caribbean islands have experienced problems related to medicine availability and prices. To tackle these problems, the OECS members founded the Eastern Caribbean Drug Service (ECDS) in 1986 after the Prime Ministers of the nine Eastern Caribbean States signed the Eastern Caribbean Drug Service Agreement. In 2000, the ECDS was renamed the OECS/Pharmaceutical Procurement Service (OECS/PPS) [2]. The primary goal of the ECDS is to pooled procure medicines and medical supplies for the public sector of the participating OECS members [3,4].

During ECDS's foundation, the United States Agency for International Development (USAID) played an important role by providing technical assistance and covering the operating costs in the early stages [4,5]. By 1989, the ECDS became self-sufficient through charging a 15% surcharge on the value of each procurement order. This percentage has gradually decreased to 9% since 2016 [5,6].

Currently, the OECS/PPS consists of nine buying member states: Anguilla, Antigua and Barbuda, British Virgin Islands, Dominica, Grenada, Montserrat, St. Kitts and Nevis, St. Lucia, St. Vincent and the Grenadines [7]. The OECS/PPS product portfolio consists of 840 items including both pharmaceutical and non-pharmaceutical products, with an estimated total value of $25.4 million in purchase orders accounted between 2015 and 2016 [6].

Essential Elements

We have identified five essential elements that significantly contributed to the establishment and current functioning of the OECS/PPS.

1. **Alignment on goals, purpose and operations of ECDS**

The Eastern Caribbean island nations faced similar problems related to medicine procurement. These problems included a small market size, limited availability of health products, relatively high costs of procurement of health products, and a limited efficiency of procurement and supply management. When problems are shared, the motivations to participate and the goals the individual buyers aim to achieve through the pooled procurement mechanism are much more likely to converge. This allowed the OECS members to set up a central contracting mechanism, the most integrated form of pooled procurement. The lack of a pharmaceutical industry at the OECS members can be seen as an additional facilitator in the process of setting up the ECDS. Taking one important stakeholder

out of the equation reduced the complexity in aligning goals and motivations between stakeholders, and it also removed the need for the OECS members to protect their national industrial interests.

During the establishment of the ECDS, the OECS members formalized their shared vision into a shared plan by articulating their goals, purpose and operations of the pooled procurement mechanism in the "Agreement Establishing the Eastern Caribbean Drug Service".

2. Homogeneity of buyer's characteristics related to their needs

As mentioned above, the needs of the buyers (i.e., OECS members) were very similar. The island nations needed to increase their market size to increase availability of health products, to contain costs and to increase procurement and supply efficiency. The characteristics of the Caribbean islands moreover is highly homogeneous, related to these shared needs. For example, the OECS members experience the collective need to increasing their market size because all OECS members share similar characteristics in population size, demographics and financial capacity. If these characteristics were greatly diverging among OECS members, this need of increasing market size might have been less relevant for some of the countries, resulting in potential conflicting interests among buyers.

High costs and limited availability of health products were other shared needs among OECS members. These needs were partially attributable to the shared characteristics of population size and geography. If, for example, there were mainland countries with large population size and easy-access supply and distribution networks involved in the pooled procurement mechanism, these mainland countries might have experienced different needs. As a result, the collective need of increasing availability and reducing costs of health products would have been less urgent for all buyers, having further implications for the goals, purpose and operations of the pooled procurement mechanism.

Other shared characteristics among OECS members include currency, cultural factors, and epidemiology, which results in demand for similar products, which is updated biennially in the *Regional Formulary and Therapeutics Manual*.

3. History of collaboration, and organizational & political structures already in place

In setting up the pooled procurement mechanism, the OECS members could benefit from the presence of pre-existing organizational and political structures. One important reason for establishing the OECS was to integrate these pre-existing organizational and political structures under a single roof. These structures include the Eastern Caribbean Currency Authority established in 1965, the predecessor of the Eastern Caribbean Central Bank (ECCB), the Directorate of Civil Aviation established in 1957; and the Eastern Caribbean Supreme Court established in 1967 [1].

Other areas of OECS collaboration that reinforce trust among the buyers include information sharing, harmonization of laws and educational standards, disaster preparedness, and natural resources management [3]. In addition, most OECS members are also member of other regional bodies, including the Caribbean Community (CARICOM), which was established in 1973 [8].

4. Predictable, timely and efficient payment mechanism

One critical element of the ECDS's success is the presence of the Eastern Caribbean Central Bank (ECCB). The ECCB allows the participating countries to pay suppliers in foreign exchange at no additional cost. While the ECDS carries out the tender and procurement

related activities, the ECCB is responsible for paying the suppliers directly, while also covering the ECDS's operating costs with the surcharge. The suppliers then ship the orders directly to the OECS members. After receiving the order, the OECS members reimburse their individual drug account at the ECCB [3,4]. Although this payment mechanism has been considered as highly efficient, late payments from OECS members to the ECCB have remained a challenge [6,9,10]. This has resulted in suppliers withholding shipments to both late and on-time paying OECS members, and a reduced participation of suppliers in OECS tenders [11].

5. **Standardized and transparent procedures**

The OECS/PPS has standardized and transparent procedures, which have been articulated by the OECS Commission in their *Procurement Manual* [12], last revised in 2016. Adhering to these procedures incentivizes both buyers and suppliers to participate in the mechanism.

The Policy Board, consisting of the OECS Ministers of Health, the OECS Director General, a representative of the ECCB and OECS/PPS Head of Unit, is responsible for approving contracts and framework agreements for pharmaceuticals [12].

There are two committees in the OECS/PPS that report to the Policy Board [12]:

1. The Technical Advisory Committee (TAC) consists of senior doctors and the Central Medical Stores (CMS) Managers of each OECS Ministry of Health, with the main responsibility of reviewing and updating the *Regional Formulary and Therapeutics Manual*.
2. The PPS Bid Committee, formerly known as the ECDS Drug Tenders Committee. Voting members of this committee are the CMS managers of each OECS member. The chairperson rotates every 18 months based on alphabetical order of the OECS members. The primary role of the committee is to ensure that PPS purchases are made in compliance with the standardized policies and procedures. The PPS Bid Committee has several sub-committees, including:
    a. Bid Opening sub-committee
    b. Internal Bid sub-committee
    c. Selection sub-committee
    d. Evaluation sub-committee

These standardized and transparent procedures also reduce potential conflict of interest or corruption. Other contributing factors include OECS/PPS staff members not being allowed to vote at the stage of awarding the contract, and the OECS providing competitive salaries compared to national-level procurement agencies, reducing the temptation of potential corruption at the OECS/PPS [4].

Essential elements/actor	Organisation of the Eastern Caribbean States (OECS)	Ref.
**A. Buyers**		
**All buyers <u>need</u> to have individually**:		
1. Perceived problem for which pooled procurement may be a solution (problem)	Small market size, limited availability of health products, relatively high costs of procurement and health products, limited efficiency of procurement and supply management.	[3,11,13]
2. Motivations that outweigh the opportunity costs	- One of the factors that contributed to the buyer's motivation to participate was the buyer's (i.e., OECS members) representation in the committees of the OECS/PPS. This allows the OECS members to actively participate in the decision-making of the pooled procurement organization, and secure the political commitment of the respective Ministries of Health by giving buyers a sense of ownership over the organization.<br>- Another contributing factor was a result of the Eastern Caribbean Central Bank (ECCB) facilitated the prompt payment of the foreign exchange to suppliers at no additional costs for the participating OECS members.	[10]
3. Budget, either internal or external (through donors)	At the initiation of the Eastern Caribbean Drug Service (ECDS), the predecessor of the OECS/PPS, the OECS members deposited one-third of their annual internal pharmaceutical budget to individual country drug accounts at the Eastern Caribbean Central Bank (ECCB) to assure prompt payment to suppliers and to maintain a revolving drug fund.<br>However, late payments from OECS members to the ECCB have remained a challenge, as explained under B6.	[10]
4. Sufficient technical capacity (e.g., demand forecasting)	Although there is some degree of technical capacity, one of the remaining challenges of the OECS/PPS is poor accuracy of demand forecasting.	[9]
5. Compatible laws, regulations and policies that allow for (international) pooled procurement	As further explained under B17, OECS members have been collaborating on multiple areas, including establishing an Eastern Caribbean Supreme Court in 1967, and further harmonization of laws.<br>However, harmonization on procurement-related laws and regulations among OECS members still seems lacking, with outdated procurement laws in some OECS members (e.g., Dominica, Grenada and St. Lucia) and even lacking laws on governing public procurement in other OECS members (e.g., Saint Vincent and Grenadines).	[3,14]
**If buyer's mechanism, all buyers combined, <u>need</u> to have**:		
6. Demonstrated willingness to solve their problem collectively through pooled procurement (shared vision)	- The first step of expressed willingness and political commitment was shown by the Prime Ministers of the Eastern Caribbean States by agreeing to establish the OECS/PPS in 1986.<br>- Another important sign of great political commitment was the financial contribution and commitment of each OECS member prior to the establishment of the ECDS, which is not common.<br>- Further political commitment from Ministries of Health was secured by setting up committees that operate based on participatory decision-making, as explained in A2.	[3,10]

7.	Alignment on goals, purpose and operations of the pooled procurement mechanism (shared plan)	The goals, purpose and operations of the pooled procurement organisation have been articulated in the *Agreement Establishing the Eastern Caribbean Drug Service* and signed by the participating OECS members in June 1989.<br>One factor that reduced the complexity of aligning goals, purpose and the operations of the ECDS was the lack of a regional pharmaceutical industry: there were no pharmaceutical manufacturers and few wholesalers based in the OECS members, and the market was too small to warrant powerful lobbying tactics by transnational companies.	[3,13]
8.	Joint need for specific products (product alignment)	OECS members agreed on the goals, purpose and operations of the pooled procurement mechanism, as explained under A7. This includes the scope of products to procure. The Technical Advisory Committee (TAC) of the OECS/PPS, further explained under B1, produces a biennial *Regional Formulary and Therapeutics (F&T) Manual*, which consists of a selection of essential medicines common to the participating OECS member states. This list of essential medicines has also significantly reduced wastage, because budget is not tied up in overstocks and non-essential items. The F&T manual is not only a medicines list, but it also contains information for prescribers on appropriate medicine use. The pooled procurement list represents large-volume items (class A and B) for which there is a consistently high demand in the OECS members.<br>The inter-country pooled procurement mechanism does not focus on procurement of other products that could be procured more efficiently through other mechanisms. For example, relatively low-use items and products with fluctuating consumption patterns such as some anti-cancer drugs are excluded from the tender. Also, biological products and vaccines which can be obtained at a low cost from the PAHO Expanded Program of Immunization have been similarly precluded from the tendering process.<br>ECDS procured approximately 85% of the public sector purchases, in 1996.	[3,10]
9.	Sufficient market size	The market size of each participating OECS members has significantly increased. The combined population of the member states that participate in the OECS/PPS program has increased to approximately one million.	[9]
10.	Sufficient and stable financial capacity	As explained under A3.	[10]
11.	Regulatory harmonization (e.g., shared quality standards, joint assessment, mutual recognition, etc.)	The OECS/PPS has implemented a prequalification mechanism for suppliers, prior to the tender. During this prequalification process, suppliers are evaluated on quality standards, technical competence, and financial viability. However, besides shared quality standards for suppliers, little is known about regulatory harmonization, including mutual recognition of Medicine Regulatory Agencies and provision of market authorization among OECS members. In St. Lucia, for example, no marketing authorization is required for pharmaceuticals to enter the market.<br>The Caribbean Public Health Agency (CARPHA), which operates under CARICOM, has recently put a mechanism in place called the Caribbean Regulatory System (CRS). In 2018, a partnership between OECS/PPS and CRS was announced on Antiretroviral (ARV) medicines. ARVs had to receive approval from CRS before suppliers were eligible to participate in the OECS/PPS ARV tender of June 2019.	[10,11,15,16]

	However, more collaboration and integration are required among OECS members to achieve regulatory harmonization.	
12. Trust (in other buyers and the pooled procurement organization)	The factors described under A17, have contributed to a positive relationship among buyers prior to the establishment of the ECDS. This generated a sufficient level of trust among buyers to set up the ECDS.<br>Other factors that contributed to increased trust between buyers and the pooled procurement organization (OECS/PPS) are:<br>1. OECS/PPS providing the OECS members with a wide range of related services, which include training and technical assistance, a common drug formulary manual, drug utilization studies and quality assurance.<br>2. Participatory decision-making, as explained under A2<br>3. Standardized and transparent procedures, as explained under B3<br>4. Minimization of conflict of interest, as explained under B10	[3,10]
13. Transparent data and information sharing	As further explained under B17, OECS members have been collaborating on multiple areas, including information and data sharing.	[3]
14. No history of conflict or failed collaboration	Existing history of collaboration within OECS has been described under A17.<br>In the early 1980s, the Caribbean Community (CARICOM) tried to establish a similar pooled procurement mechanism but failed due to a lack of political will. This might have been partially attributable to diverging characteristics of the buyer's pool, leading to diverging needs of the buyers and clashing goals, purpose and operations of the pooled procurement mechanism.	[3,11]
15. Homogeneity of buyer's characteristics related to their needs	The more homogeneous the characteristics of the buyers (i.e., OECS members) are, the more likely that there will be no conflicting needs, and the more likely that the pooled procurement mechanism will satisfy these needs.<br>The needs for the OECS members were very similar, as described under A1.<br>In the OECS case, the island nations share many similarities in characteristics related to their needs, including population size, demographics, geography, and disease profile.	
16. Shared cultural factors and values (e.g., language, traditions, etc.)	OECS members share cultural factors and values, including language and ethnicity.	[9]
17. Existing political or structural mechanisms	Prior to the OECS, there were already pre-existing organizational and political structures in place, including the Eastern Caribbean Currency Authority established in 1965, the predecessor of the Eastern Caribbean Central Bank (ECCB), the Directorate of Civil Aviation established in 1957; and the Eastern Caribbean Supreme Court established in 1967. To integrate these structures under a single roof, the OECS was established in 1981.<br>Although the partnership of the OECS members revolves mainly around economic cooperation, there has been integration and collaboration around other areas, including information sharing, harmonization of laws and educational standards, disaster preparedness, natural resources management.<br>Besides the OECS, the island nations are also collaborating in other regional political structures, including the Caribbean Community (CARICOM), which was established in 1973.	[1,3,9–11]

B. Pooled Procurement Organization

1.	Organizational structure with clear roles and responsibilities	The OECS/PPS has set up an organizational structure with clear roles and responsibilities. The appointment and roles of staff have been articulated in the *Agreement Establishing the Eastern Caribbean Drug* Service, at the initiation of the ECDS (i.e., predecessor of the OECS/PPS).<br><br>The Policy Board, consisting of the OECS Ministers of Health, the OECS Director General, a representative of the ECCB and OECS/PPS Head of Unit, is responsible for approving contracts and framework agreements for pharmaceuticals [12].<br><br>There are two committees in the OECS/PPS that report to the Policy Board [12]:<br>1. The Technical Advisory Committee (TAC), consists of senior doctors and the Central Medical Stores (CMS) Managers of each OECS Ministry of Health, with the main responsibility of reviewing and updating the *Regional Formulary and Therapeutics Manual*.<br>2. The PPS Bid Committee, formerly known as the ECDS Drug Tenders Committee. Voting members of this committee are the CMS managers of each OECS member. The chairperson rotates every 18 months based on alphabetical order of the OECS members. The primary role of the committee is to ensure that PPS purchases are made in compliance with the standardized policies and procedures. The PPS Bid Committee has several sub-committees, including:<br>e. Bid Opening sub-committee<br>f. Internal Bid sub-committee<br>g. Selection sub-committee<br>h. Evaluation sub-committee<br><br>Although the OECS/PPS is responsible to carry out tenders, awarding contracts, placing orders to suppliers and carrying out performance assessment, it is the ECCB that pays suppliers directly, further explained under B6.	[3,9,10,13]
2.	Clear mandate	Articulated and signed by OECS members in the *Agreement Establishing the Eastern Caribbean Drug Service*, at the initiation of the ECDS.	[13]

3.	Standardized and transparent procedures	The OECS/PPS has standardized and transparent procedures, which have been articulated by the OECS Commission in their *Procurement Manual*, last revised in 2016.<br>At its initiation, the Eastern Caribbean Drug Service set up standardized guidelines and procedures for their tender processes. Developing written guidelines and adhering to these guidelines has increased transparency in the OECS procurement process, increasing trust for the pooled procurement organization among both buyers and suppliers.<br>Based on OECS documents, the procurement and payment cycle of the OECS/PPS is as follows: OECS/PPS aggregates demand forecasts from OECS members; OECS/PPS invites bids from suppliers; OECS/PPS awards contract based on framework agreements; OECS members confirm demand to OECS/PPS; OECS/PPS places order directly at supplier; Supplier ships goods directly to OECS member; ECCB pays suppliers & OECS/PPS; OECS members reimburse ECCB.	[3,9,10,12]
4.	Sufficient, predictable and timely budget, either internal (through service fees) or external (through donors) to carry out pooled procurement	As described under A3, the OECS members deposited one-third of their annual internal pharmaceutical budget to individual country drug accounts at the Eastern Caribbean Central Bank (ECCB) to assure prompt payment to suppliers and to maintain a revolving drug fund.<br>However, in recent years, there has been an increase in late payments by OECS members, as explained under B6. Potential solutions to increase sufficient, predictable and timely budget proposed by OECS/PPS were to implement national health insurance, increase budgetary allocation of OECS members, match individual country expenditure with their allocated budget, and apply to external funding.	[6,10]
5.	Sufficient, predictable and timely budget, either internal (through service fees) or external (through donors), to cover organizational expenses	At initiation of ECDS in 1986, USAID provided technical assistance and covered operating costs with a grant estimated to be around US$ 3.5million. There was no external funding provided for pharmaceutical procurement.<br>Initial surcharge on the value of each procurement order was 15% between 1986-2000. It was reduced to 13% between 2001-2005, and further reduced to 11% between 2006-2015, all the way to its current level of 9% since 2016. The ECCB pays suppliers directly, while at the same time covering the OECS/PPS operating costs with the surcharge.	[3–6,9,10]
6.	Predictable, timely and efficient payment mechanism	The Eastern Caribbean Central Bank (ECCB) allows the participating countries to pay suppliers in foreign exchange at no additional cost. While the OECS/PPS carries out the tender and procurement related activities, the ECCB is responsible for paying the suppliers directly.<br>The suppliers then ship the orders directly to the OECS members. After receiving the order, the OECS members reimburse their individual drug account at the ECCB. All OECS member states are charged the same price, regardless of volume, for the duration of the contract – an important benefit for the smallest buyers.<br>Although this payment mechanism is considered as highly effective, late payments from OECS members to the ECCB have remained a challenge. In 2016, only 32% of the 590 invoices were paid within the 60-day contractual payment term. This number was 57% in 2017. This has resulted in suppliers withholding shipments to both late and on-time paying OECS members, and a reduced participation of suppliers in OECS tenders.	[3,4,9–11]

7.	Human resources (sufficient in numbers and expertise)	Prior to the establishment of the ECDS, there were concerns about the procurement expertise of staff, contributing to medicine shortages in health facilities in OECS members.<br>Due to training programs and providing competitive salaries in these procurement agencies, the number and expertise of staff has increased over the years. This number was 15 full-time equivalent (FTE) staff members for the OECS/PPS in 2007.	[4,11]
8.	Sufficient technical capacity (e.g., procurement, quality assessment, forecasting, etc.)	The technical capacity of human resources has been explained under B7.<br>Although there is no direct data on the current state of technical capacity within OECS/PPS, the OECS/PPS has developed detailed and written procurement guidelines, as explained under B3. The fact that the OECS/PPS operations are still running applying these standardized and transparent procurement processes, seems to confirm that the OECS/PPS has sufficient technical capacity.<br>As explained under A4, one of the remaining challenges of the OECS members is poor accuracy of demand forecasting. One solution might be for OECS/PPS to focus more on training staff in its member countries to accurately forecast and report demand.<br>In addition, OECS/PPS monitors and reviews supplier performance, including lead time and quality, annually. OECS members can submit oral and written quality issues they experienced with suppliers, which are then tested for confirmation at the Caribbean Regional Drug Testing Laboratory (CRDTL), located in Jamaica. The CRDTL is established as part of CARICOM. This provides another example, in addition to the example of biologicals procurement from PAHO explained under A8, of the OECS also collaborating with and relying on other regional political structures.	[2,3,9,11]
9.	Positive reputation	The historical track record of the OECS/PPS has earned them a positive reputation, and worldwide recognition as one of the primary examples of a well-functioning inter-country pooled procurement mechanisms.<br>This positive reputation has been reinforced by the OECS/PPS providing a rounded procurement service to its buyers and suppliers, in which the OECS/PPS provide OECS members with a wide range of related services, which include training and technical assistance, a common *Regional Formulary and Therapeutics* Manual, medicine utilization studies and quality assurance.<br>In recent years, however, the positive reputation for prompt payment of the OECS/PPS has decreased because of delayed reimbursement of the ECCB by the OECS members, as explained under B6.	[6,10,11]

10. No conflict of interest	The OECS/PPS has multiple processes in place to minimize the possibility of conflict of interest or corruption in its procurement processes:<br>1. Standardized and transparent procurement processes, as explained under B3;<br>2. Mandatory disclosure of interest by OECS/PPS Policy Board members;<br>3. The collective decision-making of OECS members in the Tenders sub-committees of OECS/PPS reduces the possible influence of the pharmaceutical industry. This is reinforced by a lack of a regional pharmaceutical industry based in the OECS members, reducing lobbying of industry representatives;<br>4. Lack of incentives for OECS members to favor national suppliers, because there are no domestic manufacturers in the region, as explained under A7;<br>5. The opening of tenders offers takes place publicly with staff members from multiple OECS/PPS committees being present;<br>6. The OECS/PPS staff members are not allowed to vote at the stage of awarding the contract;<br>7. Rotating chair of the PPS Bid Committee among the OECS members, as explained under B1<br>8. Providing competitive salaries to OECS/PPS staff, as explained under B7, which reduces temptations of corruption.	[3,4,10,13]
11. User-friendliness (both towards buyers and sellers)	The standardized procurement guidelines, as explained under B3, contribute to a transparent and user-friendly procurement process. In addition, OECS established an electronic public procurement system (e-PPS) to facilitate a transparent and user-friendly tendering process. Also, the facilitation of payments by the Eastern Caribbean Central Bank, as explained under B6, has increased the user-friendliness of financial transaction between buyers, the organization and suppliers.<br>However, information on individual OECS member or supplier experiences and satisfaction with the OECS/PPS is lacking.	[17]
**C. Suppliers**		
1. Sufficient number of qualified suppliers	The OECS/PPS award individual contracts for each product to a primary and a secondary supplier. This mechanism can safeguard the supply when the primary supplier is not able to fulfil its contractual obligations. Involving the secondary supplier in this case will also avoid the delay that would've been caused by a rebidding process of suppliers. This mechanism is only expected to operate as intended if the price of the secondary supplier is relatively close to the primary supplier, and the secondary supplier is willing to cover the contractual terms in case the primary supplier is not able to fulfil. Unless the OECS/PPS has concerns regarding quality or performance of the supplier, the lowest bidder often receives the primary award.<br>We found no published information on the number of qualified suppliers that participate in OECS/PPS tenders.	[3,11]

2. Sufficient production incentives	Less relevant for OECS/PPS, because the OECS/PPS is procuring high demand essential medicines that are frequently being consumed and produced. Supply incentives, outlined under C3, are more relevant for OECS/PPS. Production incentives are expected to be more relevant for disease specific, product specific or single source pooled procurement mechanisms, that aim to create a market for products that are not being produced, or not in sufficient quantities.	
3. Sufficient supply incentives	The OECS/PPS provides multiple supply incentives for suppliers to supply to the OECS region:<br>1. Standardized and transparent procurement guidelines, as explained under B3;<br>2. A centralized financing mechanism that is being managed by the ECCB, as explained under B6. This mechanism allows suppliers to be paid directly, and provides suppliers with a central point of contact in case of late payments of individual buyers;<br>3. A generally positive reputation, as explained under B9<br>4. A guarantee of the public sector market in the OECS members, because the OECS members have agreed on exclusively procuring the products that are being tendered through the OECS/PPS mechanism. As a result, non-awarded suppliers could not undercut the price by deducting the 9-15% surcharge fee for OECS/PPS operational costs through supplying outside of the OECS/PPS, directly to OECS members. However, there have been recent reports of OECS members purchasing outside of the OECS/PPS;<br>5. A consolidated and sufficient market size, as described under A9;<br>6. 18-month framework agreements, once a supplier is awarded the contract by the OECS/PPS;<br>7. Primary and secondary supplier contracts, as explained under C1	[3,7,9,10,18]
4. Sufficient number of distributors with favourable delivery terms	Due to its geographical location, OECS members have experienced problems with favourable distribution and supply terms.<br>Approximately 90% of the items procured by ECDS (i.e., now OECS/PPS) has been through air travel, because compared to sea travel, air travel is more reliable, much faster, easier port clearance and price differences for most items relatively similar. Sea travel is mainly used for IV fluids, due to its relatively large volume and weight. Within OECS/PPS, all tender prices are CIF (cost, insurance and freight) for both air and sea travel. Under CIF incoterms, the seller covers the costs up to arrival at named port or destination, including insurance. Standardizing this allows for better comparisons between suppliers during the tender process. CIF has been mentioned as the best option for the OECS/PPS due to the relatively high transportation costs for the small shipments to each individual OECS member.	[3,11]

References

1. OECS Secretariat. Economic Union Treaty - Frequently Asked Questions (FAQs). 2008. Available from: http://ctrc.sice.oas.org/CARICOM/OECS/FAQs_OECS_Integration.pdf

2. Pan American Health Organization. Pharmaceutical Situation in the Caribbean: Fact book on Level 1 Monitoring Indicators - 2007. Washington, D.C.: PAHO.; 2010.

3. Huff-Rousselle M, Burnett F. Cost containment through pharmaceutical procurement: A Caribbean case study. Int J Health Plann Manage. 1996;11:135–57.

4. Huff-Rousselle M. The logical underpinnings and benefits of pooled pharmaceutical procurement: a pragmatic role for our public institutions? Soc Sci Med. 2012;75:1572–80.

5. World Health Organization. Regional workshop on strengthening quantification and procurement of essential medicines. Report of the workshop held in New Delhi, India, 10-12 June 2014. 2014; Available from: https://apps.who.int/iris/handle/10665/206213

6. OECS/PPS. 30th OECS/PPS Policy Board meeting. Dominica; 2016. Available from: https://www.oecs.org/en/our-work/knowledge/library/pps-annual-report-2016/viewdocument/680

7. Macé C. Pooled Procurement of Insulin and Associated Supplies. Health Action International; 2022. Available from: https://haiweb.org/wp-content/uploads/2022/02/Pooled_Procurement_of_Insulin.pdf

8. Gasiorek M, Haynes-Prempeh M. Part 2: The Caribbean region and the OECS: an overview. 2006. Available from: https://assets.publishing.service.gov.uk/media/57a08c1c40f0b64974000fd2/EPACaribbeanFinalReport2.pdf

9. Burnett F. Improving Access to NCDs medicines. Washington D.C.; 2017. Available from: https://www.paho.org/hq/index.php?option=com_docman&view=download&category_slug=increasing-access-to-ncd-medicines-caribbean-22-23-february-2017-9056&alias=38580-improving-access-to-ncds-medicines-oecs-580&Itemid=270&lang=en

10. Burnett F. Reducing costs through regional pooled procurement. Essential Drugs Monitor. 2003;32:7–8.

11. Management Science for Health. MDS-3: Managing Access to Medicines and Health Technologies (Third Edition). Arlington, VA: Management Science for Health; 2012. Available from: http://apps.who.int/medicinedocs/documents/s19577en/s19577en.pdf

12. OECS Commission. Procurement Manual. 2016. Available from: https://www.oecs.org/en/work-with-us/procurements/procurement-procedures/procurement-manual-revised-june-2016/download

13. OECS. Agreement Establishing the Eastern Caribbean Drug Service. ECDS; 1990. Available from: http://ctrc.sice.oas.org/CARICOM/OECS/ECDS.pdf

14. The World Bank. Project Information Document / Identification /Concept Stage (PID). 2018 Mar. Report No.: PIDC138590. Available from: http://documents1.worldbank.org.eur.idm.oclc.org/curated/pt/523131520345936167/pdf/Project-Information-Document-PID-OECS-Public-Procurement-Network-P166039.pdf

15. CARPHA/CRS and The Organization of Eastern Caribbean States/Pharmaceutical Procurement Service Form Partnership. Caribbean Public Health Agency. 2018. Available from: https://carpha.org/More/Media/Articles/ArticleID/223/CARPHA-CRS-and-The-Organization-of-Eastern-Caribbean-States-Pharmaceutical-Procurement-Service-Form-Partnership

16. Ministry of Health, Wellness, Human Services and Gender Relations, PAHO/WHO. Saint Lucia Pharmaceutical Country Profile. 2012. Available from: https://www.paho.org/hq/dmdocuments/2013/PHARMACEUTICAL-COUNTRY-PROFILE-SAINT-LUCIA.pdf

17. Minto-Coy ID, Berman E. Public Administration and Policy in the Caribbean. CRC Press; 2015.

18. Nemzoff C, Chalkidou K, Over M. Aggregating Demand for Pharmaceuticals is Appealing, but Pooling Is Not a Panacea. Center for Global Development; 2019 May. Available from: https://www.cgdev.org/sites/default/files/aggregating-demand-pharmaceuticals-appealing-pooling-not-panacea.pdf
