# Supplementary material for: Does structural form matter? A comparative analysis of pooled procurement mechanisms for health commodities
Source: Global Health. 2023 Nov 23;19:90. doi: 10.1186/s12992-023-00974-1 (PMC10668364; doi:10.1186/s12992-023-00974-1)
Supplement: Supplementary file 2 — Additional file 2. [file 12992_2023_974_MOESM2_ESM.pdf]

Creation stage – Pacific Island Countries (PIC)

General characteristics and historical developments

The Pacific Island Countries (PIC) consist of a broad range of island nations in the Pacific Ocean. These islands have generally been subdivided into three main groups: Micronesia, Melanesia and Polynesia. These groups are not only divided in geographic location, but also in ethnicity, language, culture, and traditions [1]. Table 1 shows the great variety in some geographic, demographic and economic characteristics between the Pacific Island Countries. When we refer to the Pacific Island Countries, we use the term flexibly to refer to the island nations of the Pacific region that are involved in discussions on inter-country pooled procurement within that particular timeframe and context.

**Groups**	**Pacific Island Country**	**Land** (km^2)	**Population**	**Currency**	**GDP/capita** (USD)	**Current health expenditure** (% of GDP)
**Melanesia**	Fiji	18,333	884,887	FJD	6,152	3.42
	New Caledonia	18,576	271,407	XPF	37,448	-
	Papua New Guinea	462,840	7,275,324	KINA	2,854	2.37
	Solomon Islands	28,230	515,870	SBD	2,295	4.47
	Vanuatu	12,281	272,459	VUV	3,260	3.37
**Micronesia**	Federated States of Micronesia	701	102,843	USD	3,830	12.59
	Guam	541	159,358	USD	34,153	-
	Kiribati	811	110,136	AUD	1,636	12.11
	Marshall Islands	181	53,158	USD	4,337	17.55
	Nauru	21	11,550	AUD	11,666	9.58
	Northern Mariana Islands	457	53,883	USD	23,550	-
	Palau	444	17,661	USD	15,673	10.87
**Polynesia**	American Samoa	199	55,519	USD	11,245	-
	Cook Islands	237	14,802	NZD	24,913	-
	French Polynesia	3,521	275,898	XPF	22,308	-
	Niue	259	1,591	NZD	18,756	-
	Pitcairn Islands	49	57	NZD	3,056	-
	Samoa	2,934	195,979	SAT	4,284	5.21
	Tokelau	12	1,499	NZD	6,882	-
	Tonga	749	100,651	TOP	5,081	5.10
	Tuvalu	26	10,566	AUD	4,223	19.05
	Wallis and Futuna	142	11,558	XPF	11,674	-

Table 1. Geographic, Demographic and Economic Characteristics of Pacific Island Countries. Sources: [2–4].

The South Pacific Forum, now called the Pacific Islands Forum, was established in 1971, with the participation of the heads of state of seven Pacific Island Countries. Since then, it has developed into the region's leading political and economic collaboration initiative. It aims include to increase cooperation among member states and enhance economic development [5,6]. Currently, the Forum has 13 full members, after the recent withdrawal of the Micronesian sub-group (i.e., Palau, Marshall Islands, the Federated States of Micronesia, Kiribati, and Nauru) over a dispute on selecting the Forum's new Director-General [7].

Discussions about inter-country pooled procurement in the Pacific region have been going on since the 1970s. In December 1978, the World Health Organization (WHO) co-organized a meeting in Fiji on a harmonized essential medicines list and discussions were held on the possibility of a bulk purchasing scheme for medical supplies [8].

The year after, the 32nd World Health Assembly in May 1979 mentioned that several preliminary surveys in the Western Pacific Region were carried out, a regional medicines list was adopted and proposed to establish a regional pharmaceutical service to manage procurement, quality assessment, storage and distribution [9].

Later that year in August, the WHO co-organized the Working Group on Technical Cooperation in Pharmaceutical Supplies in the South Pacific. The working group recommended to establish the **South Pacific Pharmaceutical Service**, that would include a Joint Purchasing Service [10]. The region's Ministers of Health confirmed and declared their intent to establish the South Pacific Pharmaceutical Service during a conference in Manila, Philippines in November 1979 [11].

Due to difficulties in creating necessary preconditions, the **South Pacific Pharmaceutical Service** was never established [12]. Pacific countries could not align on the specific products and sources, procurement and financial cycles, location for its headquarters and payment mechanisms, including which currency to use [13,14]. Instead, the **South Pacific Pharmaceutical Project** was established in 1984. The goal of this project was to provide Pacific countries advisory services through a WHO pharmacist and exchange information on medicines, which could then potentially be developed into a pooled procurement mechanism [5,13].

Meanwhile, the government of Fiji established the **Fiji Pharmaceutical Service Bulk Purchasing Scheme** (FPS/BPS) in 1981. The purpose of this scheme was to supply pharmaceutical products to Fiji's public and private health facilities, as well as to several smaller island states (SIS) of the Pacific [5,15]. The FPS/BPS is still operational, but used limitedly by other PIC due to several challenges. While some SIS experienced problems with poor communication and logistical constraints, Fiji expressed concerns about delayed payments [16].

In March 1995, the Pacific Island Countries (PIC) signed the **Yanuca Declaration**. The declaration stated that the countries agreed to "establish a multidisciplinary committee to further analyse the benefits of establishing a bulk purchasing scheme" [17].

A year later, the WHO facilitated a feasibility study on pooled procurement in the region. The report suggested an inter-country pooled procurement mechanism that would supply directly to its member countries. However, the study failed to provide details on its rationale for its decision, the products to be procured, and the mechanism's operations [5].
The following year, in 1997, the PIC signed the **Rarotonga Agreement Towards Healthy Islands**. In this agreement, the Ministers of Health of 17 PIC agreed "to identify and take action to overcome obstacles to the implementation of bulk purchasing schemes for *pharmaceuticals* and other health supplies, and to address related issues such as quality assurance and drug information exchange, through agreements between interested countries in the Pacific"[18].

In March 1999, the Pacific Island Countries signed the **Palau Action Statement,** in which they reaffirmed their earlier recommendations of encouraging collaboration between countries on pooled procurement of pharmaceuticals [19].

Although there have been a few annual meetings and working groups in the years after, little was achieved in setting up an inter-country pooled procurement mechanism. According to another feasibility study described below [5], the reason was that certain criteria to set up an inter-country pooled procurement mechanism for medicines were not present in the region. These criteria were a lack of information on medicine needs, the need for additional infrastructure building and the lack of financial commitment by the buyer countries. Therefore, Pacific island countries have remained to "encourage" pooled procurement, instead of committing to it as a tangible goal to be "achieved" by adopting a clear roadmap. Pooled procurement lost its priority during subsequent ministerial meetings, and was mainly brought up when cost containment or quality issues were raised.

Then, as part of collaborative initiative between WHO and the European Commission, referred to as the "EC/ACP/WHO Partnership on Pharmaceutical Policies", several meetings were organized in the Pacific region [20]. During one of these meetings, the Workshop on Pharmaceutical Policies and Access to Good Quality Essential Medicines for Pacific Island Countries in Fiji in August 2006, the discussions on pooled procurement of pharmaceuticals in the PIC resurfaced, based on two recent studies published on pooled procurement of health products. This meeting was followed by multiple meetings in the Region and a suggestion by PIC to conduct another feasibility study on pooled procurement by the WHO [16,20,21].

In 2007, this WHO feasibility study was conducted. The study explored three options for an inter-country pooled procurement mechanism in the PIC region: to expand the activities of the FPS/BPS using central contracting (in line with the "Organisation of the Eastern Caribbean States example"), to establish a new pooled procurement mechanism and include the relatively big PIC (e.g., Fiji, Samoa Solomon Islands, Tonga, Vanuatu) using group contracting (in line with the "Gulf Cooperation Council example"), and finally a hybrid between these two options where smaller island states (SIS) can have the option to procure through either the inter-country pooled procurement mechanism or the FPS/BPS, where Fiji would represent the interests of the SIS [5]. During the Informal Consultation on Pooled Procurement of Essential Medicines for Pacific Island Countries in Tonga in August 2007,

participants agreed that the group contracting options would be the most feasible option for the Pacific Island Countries [16].

However, during the Eighth Meeting of Ministers of Health for the Pacific Island Countries in Madang, Papua Guinea, in 2009, the Ministers of Health of the Pacific Island Countries expressed reservations about endorsing phase one of the roadmap (i.e., harmonization and standardization) due to “insufficient information provided in the briefing document, ongoing restructuring of the medicines supply organization, decentralization of medicines procurement function in some countries, and no involvement of government decision-makers in the earlier consultation on this matter.” [22] According to one report [15], this reservation was because of health officials fear of loss of sovereignty.

There have been no further significant developments on inter-country pooled procurement of medicines in the Pacific region since.

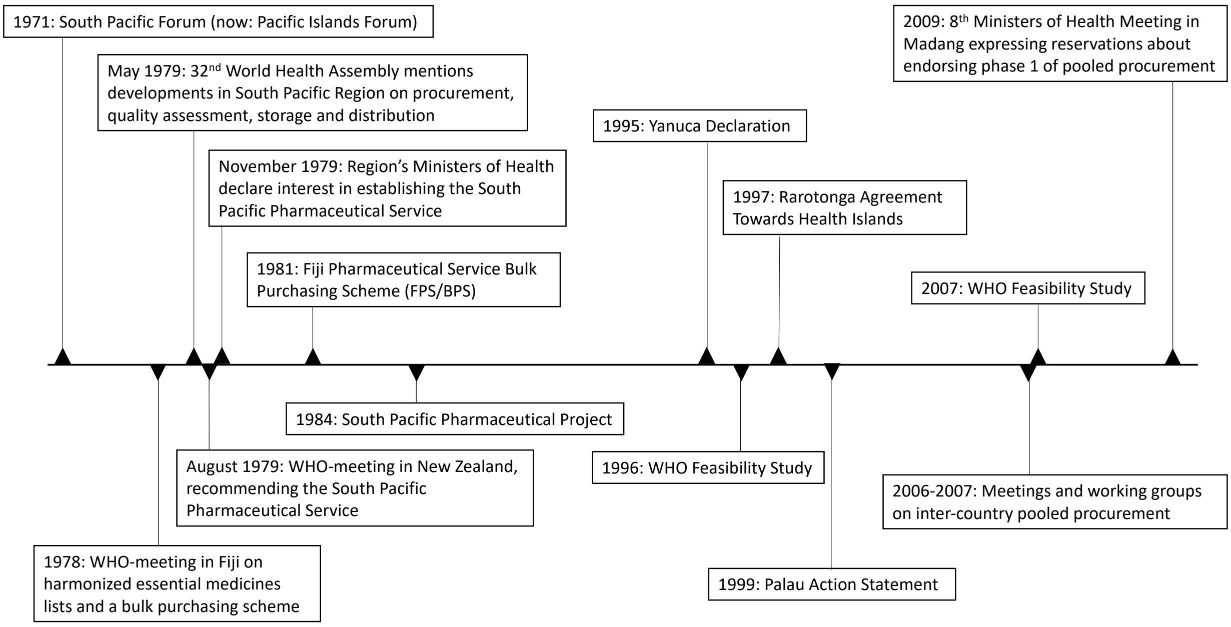

Figure 1. Timeline of events in the Pacific Island Countries.

Essential Elements

We have identified several essential elements that have not been met in setting up an inter-country pooled procurement mechanism in the Pacific Island Countries (PIC).

1. **Misalignment on goals, purpose and operations of the pooled procurement mechanism**

As discussed above, the talks about an inter-country pooled procurement mechanism for medicine have been ongoing for more than 40 years in the Pacific Island Countries. However, during these discussions, the PIC have not been able to reach alignment on goals, purpose and operations of the pooled procurement mechanism. To illustrate the misalignment in PIC's motivations to participate, several concrete examples have been provided in the literature. The motivations for small island states (SIS) were mainly to increase access to affordable medicines by increasing their market size, whereas Fiji's driving force to participate was mainly to become the leading country of the inter-country pooled procurement mechanism, expressing concerns about their sovereignty if they were not provided with this leading role [15]. However, other PIC, such as the Solomon Islands have resisted the idea of Fiji taking a leading role in the inter-country pooled procurement mechanism, partially because of past experiences of failed collaborations. For example, the attempt in 1971 to set up a joint airline, called Air Pacific. This collaboration fell apart because many PIC, particularly the Solomon Islands, believed that the airline was mainly benefitting Fiji [23]. Although there have been successful examples of collaboration initiatives in the Pacific region, such as the Forum Fisheries Agency and the South Pacific Tourism Organisation [23], the negative experiences have made PIC reluctant to rely too much on others within the inter-country pooled procurement mechanism. Inevitably, these failed collaborations have impacted the level of trust between Pacific Island Countries. Also, the great diversity in culture, tradition and languages between the PIC might have had an impact on their trust levels [1]. The fragility of the trust level between PIC were underlined recently, when five Pacific Island Countries quit the Forum over a dispute on selecting the Forum's new Director-General [7]. This may influence future efforts and discussion of an inter-country pooled procurement mechanism.

2. **Heterogeneity of buyer's characteristics related to their needs**

One of the underlying reasons for the PIC's misaligned goals, purpose and operations originates from the heterogeneity of buyer's characteristics related to their needs. Although all PIC are island nations sharing similar characteristics in geography, resulting in similar needs regarding supply chain and distribution of medicines, the PIC are greatly divergent on other characteristics. This divergence is particularly visible when we compare the PIC's population size, demographics, financial capacity, currency, and health outcomes, which might translate in different needs of health products, increasing the difficulty of product alignment [2,3,24]. We believe that this heterogeneity in health outcomes is one of the primary reasons why the PIC have not been able to achieve a harmonized essential medicines list, despite multiple attempts [16,21,25].

3. **Motivations that do not outweigh the opportunity costs**

Another underlying reason for the misalignment between PIC stems from the belief that the motivations to participate do not outweigh the opportunity costs. According to several PIC, selecting Fiji as the central hub for the pooled procurement mechanism would still result in

additional shipping costs from Fiji to other PIC [15]. In addition to distribution costs, PIC have expressed their concerns that costs of administration, and political and bureaucratic integration would outweigh the potential financial benefits gained through the inter-country pooled procurement mechanism [26].
Also, some Pacific Island Countries have had a strong historical relationship with other countries in the Region that have not expressed interest to participate in the mechanism. For example, Nauru and Kiribati, which traditionally have had strong ties to Australia, have mainly been procuring their medicines from Australia. Samoa and the Cook Islands, which have a historical connection to New Zealand, might want to procure some of their medicine from New Zealand [16]. Similarly, the PIC in the northern Pacific region (i.e., Palau, the Marshall Islands and the Federated States of Micronesia) mainly procure their medicines from Hawaii [27]. If the products procured by these Pacific Island Countries from Australia, New Zealand or Hawaii are more economical, then these PIC might compete with the inter-country pooled procurement mechanism, undermining its purpose.

4. **Lack of technical capacity and regulatory harmonization**

The Pacific Island Countries face significant shortages of technical capacity, including human resources and laboratories, to participate in an inter-country pooled procurement mechanism [5,28,29]. In addition, regulatory assessment of medicines and suppliers is practically non-existent in most PIC. Other than Fiji's Pharmaceutical and Biomedical Services, there are no national regulatory authorities present in the PIC that have expressed interest in participating in the inter-country pooled procurement mechanism [29]. All PIC lack the financial and human resources to independently set up a regulatory authority [5]. This lack of technical capacity on the individual country level is likely to carry over to the regional level, making the establishment of an inter-country mechanism more difficult. Although there have been recent examples of regulatory collaboration initiatives in the Pacific region, including several online information platforms such as "medqualityassurance.org" to share information on suppliers and products in the Pacific region [28], and PIEMEDS to share information on prices of medicines[30], the technical capacity has not been sufficient to asses product quality and register products. Therefore, before a successful inter-country pooled procurement mechanism can be set up, it would be necessary to increase technical capacity in the PIC. The support of international organizations (e.g., WHO, international procurement agencies, regulatory bodies, etc.) or other countries in the region with higher level of technical capacity (e.g., Australia, New Zealand, Singapore, Japan, etc.) can facilitate capacity building in the region.

5. **Lack of sufficient supply incentives**

Mainly due to its geographic location, market size and financial capacity, the PIC have not been able to create the circumstances to provide sufficient supply incentives for suppliers. The remote and dispersed geographical location of the island countries results in high distribution costs. The relatively small market size of the PIC, both in terms of population and value, makes it unattractive for suppliers to bear the costs of distribution, while the limited financial capacity of Pacific Island Countries limits the buyer countries to pay for the costs of distribution. In addition, the prices of medicines in the PIC are already considered relatively low, reducing the incentives for suppliers to supply the region [5]. For example, the prices of medicines in the Solomon Islands and Vanuatu were already at 90% of the prices

achieved through the pooled procurement mechanism of the Organisation of the Eastern Caribbean States.

Furthermore, the great variety in currency, a lack of a central financial institution and the lack of a central payment mechanism in the Pacific Region that can facilitate prompt payment of suppliers reduces the incentives for suppliers to supply to the Pacific region, even if an inter-country pooled procurement mechanism had been set up [5].

Finally, the lack of regulatory authorities, regulatory harmonization and a centralized registration system, which if in place, would reduce the burden for suppliers to register products in each Pacific Island Country individually, currently withholds suppliers to register their products, because the costs and efforts of registering outweigh their benefit.

Essential elements/actor	*Pacific Island Countries (PIC)*	References
**A. Buyers**		
**All buyers <u>need</u> to have individually**:		
1. Perceived problem for which pooled procurement may be a solution (problem)	Small market size, supply sustainability issues (e.g., long lead times, frequent stock-outs), lacking capacity to assess medicine quality, limited financial capacity; shortage of sufficient and qualified human resources; lacking supplier's incentives to supply and register.	[5,23,31]
2. Motivations that outweigh the opportunity costs	There have been concerns among the Pacific Island Countries (PIC) that have expressed potential interest in setting up an inter-country pooled procurement mechanism that selecting a central distribution hub, such as Fiji, will still result in additional shipping costs from Fiji to other PIC countries.<br>In addition, PIC have expressed concerns that the costs of freight, administration, and political and bureaucratic integration would outweigh the benefits of the pooled procurement mechanism	[15,26]
3. Budget to buy medicines, either internal or external (through donors)	Although recent numbers are lacking, there have been various meetings and studies where the lack of fiscal stability and shortages of financial resources for health and pharmaceutical expenditure in PIC have been mentioned. Average annual pharmaceutical expenditure between 2010 and 2014 of Fiji, Samoa, Tonga and the Solomon Islands combined was estimated to be around US$ 8.1 million.	[5,15,16,26,32]
4. Sufficient technical capacity (e.g., demand forecasting)	The Pacific Island Countries face significant shortages of technical capacity, including human resources, to participate independently in an inter-country pooled procurement mechanism. Some of these shortages are discussed under A11. Staff in the region consists often only one or two pharmacists that are responsible for the majority of tasks, including procurement and regulation while simultaneously resuming their role as hospital pharmacists. Often with little experience in procurement and supply chain management. Little is known about the capacity of demand forecasting in the Pacific Island Countries, but we expect, based on the technical capacity of PIC in other areas, that PIC are facing challenges in the accuracy of demand planning. We recommend as above capacity building from international organizations (e.g., WHO, international procurement agencies, regulatory bodies, etc.) or other countries in the region with higher level of technical capacity (e.g., Australia, New Zealand, etc.), before establishing an inter-country pooled procurement mechanism.<br>For a more in-depth review of national-level procurement processes of five selected PIC (i.e., Fiji, FSM, Kiribati, Tonga, and Tuvalu), we recommend a recent case-study by Macé [31] as further reading.	[5,31]
5. Compatible laws, regulations and policies that allow for (international) pooled procurement	The difficulty of harmonizing regulations and treatment guidelines among Pacific Island Countries has been mentioned as an important reason for the inter-country pooled procurement mechanism to not progress further. Another study on Assistive Technology in the Pacific region [33] pointed out that six of the eight Pacific Island Countries whose procurement regulations were reviewed follow World Bank procurement guidelines, and two follow the United States federal procurement guidelines. Harmonizing the procurement guidelines between PIC is necessary prior to setting up an inter-country pooled procurement mechanism.	[33,34]

If buyer's mechanism, all buyers combined, <u>need</u> to have:

6. Demonstrated willingness to solve their problem collectively through pooled procurement (shared vision)	1. As discussed above, pooled procurement of medicines has been an ongoing discussion for several decades in the Pacific Island Countries. In recent years, multiple Pacific Island Countries have expressed willingness to discover the possibilities of an inter-country pooled procurement mechanism. These possibilities have been discussed during multiple meetings, which were initiated by the World Health Organization and the European Commission, under their "EC/ACP/WHO Partnership on Pharmaceutical Policies" project:<br>a. Workshop on Pharmaceutical Policies and Access to Good Quality Essential Medicines for Pacific Island Countries in Fiji, 30 August - 1 September 2006.<br>i. Participating PIC: Cook Islands, Fiji, Kiribati, the Marshall Islands, Nauru, Palau, Papua New Guinea, Samoa, Solomon Islands, Tonga and Vanuatu.<br>b. Informal Consultation on Pooled Procurement of Pharmaceuticals for Pacific Island Countries in Fiji in 29-30 March 2007.<br>i. Participating PIC: Fiji, Samoa, Solomon Islands and Tonga<br>c. Informal Consultation on Pooled Procurement of Essential Medicines for Pacific Island Countries in Tonga, 6 August 2007.<br>i. Participating PIC: Cook Islands, Fiji, Kiribati, Nauru, Samoa, Solomon Islands, Tonga, Tuvalu and Vanuatu<br>d. Workshop on Pharmaceutical Policies, Intellectual Property Rights and Access to Essential Medicines for Pacific Island Countries in Tonga, 7-9 August 2007.<br>i. Participating PIC: Australia, Cook Islands, Fiji, Kiribati, the Marshall Islands, the Federated States of Micronesia, Nauru, New Zealand, Niue, Palau, Papua New Guinea, Samoa, Solomon Islands, Tonga, Tuvalu and Vanuatu<br>2. According to a feasibility study on pooled procurement in the Pacific Island Countries, mainly five countries (i.e., Fiji, Samoa, Solomon Islands, Tonga, and Vanuatu) have expressed interest in an inter-country pooled procurement mechanism.<br>3. However, during a Ministers of Health meeting in Madang, Papua Guinea, in 2009, the Ministers of Health of the Pacific Island Countries expressed reservations about endorsing Phase 1 (i.e., harmonization and standardization) due to insufficient information provided in the briefing instrument. According to one report [15], this reservation was because of health officials fear of loss of sovereignty. There have been no further significant developments on inter-country pooled procurement of medicines since then.	[15,16,20–23,31]

7.	Alignment on goals, purpose and operations of the pooled procurement mechanism (shared plan)	As discussed under A6, there have been multiple meetings between PIC to discuss and explore the possibilities of aligning goals, purpose and operations of the inter-country pooled procurement mechanism. However, alignment between PIC have not been reached during these meetings. Different reasons for the failed alignment have been provided:<br>- Fiji's driving force to participate was to become the leading country of the inter-country pooled procurement mechanism, and that they would be concerned about their sovereignty if they were not given this leading role;<br>- Samoa and the Cook Islands, which have had a strong historical relationship with New Zealand, might want to procure their goods from New Zealand. Other PIC, such as Nauru and Kiribati, have mainly been procuring their medicines from Australia. Procuring outside the pool (i.e., maverick buying) might compete with the PIC pooled procurement mechanism, undermining its purpose;<br>- As expressed under A2, PIC expressed concerns that selecting Fiji as a central hub would still result in in additional shipping costs from Fiji to other PIC countries;<br>- As expressed under A6, the Minister of Health of the PIC rejected the implementation of Phase 1 due to inadequate information and consultation of the PIC;<br>- These efforts to align on goals, purpose and operations of the pooled procurement mechanism have been further influenced by the history of collaboration, as expressed under A14.	[15,16,23]
8.	Joint need for specific products (product alignment)	1. During the Informal Consultation on Pooled Procurement of Pharmaceuticals for Pacific Island Countries in Fiji in March 2007, PIC agreed to harmonize their essential medicines lists before the Tonga meeting later that same year.<br>2. During Informal Consultation on Pooled Procurement of Essential Medicines for Pacific Island Countries in Tonga in August 2007, it became clear that the Pacific Island Countries had not managed to harmonize their essential medicines lists. The outcome of the Tonga meeting was that the PIC would distribute electronic copies of their EMLs and identify common essential medicines between PIC in the following six months.<br>3. Although there have been initiatives to share information and data on medicine prices and essential medicines lists, also discussed under A13, there have been no harmonized essential medicines lists set up between the PIC.	[16,21,25]
9.	Sufficient market size	During a feasibility study in 2007, it was estimated that the total market size was around US$ 21 million.<br>As expressed under A3, the average annual expenditure of 4 PIC combined was estimated to be around US$ 8.1 million.<br>Even after pooling, the value of the aggregated pharmaceutical market in the PIC has been considered as small, limiting its possibility to attract additional suppliers offering more competitive prices. Therefore, reaching economies of scale as a result of pooling is expected to have very limited financial benefits for the PIC.	[5,15]

10. Sufficient and stable financial capacity	As discussed under A3, the lack of financial stability and shortages of financial resources in PIC have been expressed.<br>In addition, concerns have been expressed about the PIC's finance departments to agree and coordinate their funding for the inter-country pooled procurement mechanism. This might be the result of a lack of a common currency among PIC, the absence of an appropriate and trusted financial organization in the region that can coordinate this process.	[5,15]
11. Regulatory harmonization (e.g., shared quality standards, joint assessment, mutual recognition, etc.)	Regulatory systems are largely informal or non-existent in the Pacific island countries. Fiji has a national regulatory authority in place (i.e., Fiji Pharmaceutical and Biomedical Services, Ministry of Health).<br>However, none of the PIC have an autonomous regulatory authority in place. Also, all PIC lack the financial and human resources to establish an autonomous regulatory authority. As a result, Pacific island countries lack a system for pharmaceutical registration. Another reason why registering products in the PIC has been difficult is due to lack of supplier incentives to register, due to the small return on investment.<br>In addition, none of the PIC interested in inter-country pooled procurement have a quality control laboratory in place. Some PIC have been sending samples to the Therapeutic Goods Administration (TGA) in Australia for testing. Bearing the costs for these tests has been difficult for some PIC. Another quality control laboratory is located in Papua New Guinea.<br>Recently, the PIC have agreed to intensify regulatory collaboration. One specific example of this collaboration has been establishing an information platform (medqualityassurance.org) with the help of the World Health Organization. The purpose of this platform is to share information on suppliers and products that would facilitate the process of regulatory assessment.	[5,28,29]
12. Trust (in other buyers and the pooled procurement organization)	Previous collaboration initiatives, as discussed under A14, have influenced the trust levels between Pacific Island Countries. Although there have been successful examples of collaboration initiatives in the region, the negative experiences have made PIC reluctant to rely too much on others in the inter-country pooled procurement mechanism.<br>Recent development, discussed under A17, have negatively impacted trust levels between the Pacific Island Countries. This may influence future efforts and discussion of an inter-country pooled procurement mechanism.	
13. Transparent data and information sharing	The Western Pacific Region has set up a platform to share information on prices of medicines. This platform, called PIEMEDS (Price Information Exchange for Selected Medicines in the Western Pacific Region) has facilitated transparent data and information sharing within Western Pacific Region, including the Pacific Island Countries. Pacific Island Countries can use this information during price negotiations with suppliers.<br>As discussed under A11, the PIC have established an online platform (medqualityassurance.org) to increase data and information sharing between the countries on regulatory assessment.	[28,30]

14. No history of conflict or failed collaboration	- As further explained under A17, the Fiji Pharmaceutical Services/Bulk Purchasing Scheme (FPS/BPS) was established in 1981. Discussions have been in place to develop the FPS/BPS into the managing organization of the inter-country pooled procurement mechanism. However, this idea has been met with resistance from other Pacific Island countries. According some accounts, particularly the Solomon Islands have resisted this idea, as a result of previous experiences with Fiji's leading role in regional collaboration initiatives. A good example of this was in the case of Air Pacific, where PIC thought that Air Pacific was mainly benefiting Fiji. The Solomon Islands were particularly discontented when Air Pacific stopped flights to Honiara in 1974 to increase its profits without consulting the Solomon Islands, cutting the Solomon Islands' air-travel connection to other countries in the region.<br>As discussed under A2, other PIC have also expressed concerns in additional shipping and distribution costs if Fiji is selected as central hub, in particular islands that are geographically furthest distanced from Fiji. On the other hand, Fiji's driving force to participate was to become the leading country of the inter-country pooled procurement mechanism, as discussed under A7.<br>- The UNICEF Vaccine Independence Initiative (VII) has been set up in 1991 to provide vaccines to selected low- and middle-income countries, including PIC. The process is as follows: UNICEF procures vaccines annually on behalf of PIC; suppliers ship orders to a cold storage facility in Fiji; vaccines are distributed to other PIC from Fiji. The UNICEF VII has had varying degrees of success in the Pacific region, with late payments from PIC and delayed shipments.<br>- In addition, the Pacific Island Countries have set up many collaboration initiatives outside the health sector. These collaboration initiatives include Pacific Islands Trade and Invest (PT&I), Forum Fisheries Agency and the South Pacific Tourism Organisation. However, there have also been collaboration initiatives that have been less successful, including Air Pacific, Pacific Forum Line and the Pacific petroleum project.	[5,15,23,31,35]
15. Homogeneity of buyer's characteristics related to their needs	Although all PIC are island countries, sharing similar characteristics in geography, resulting in similar needs regarding supply chain and distribution of medicines, the PIC are greatly divergent on other characteristics:<br>1. The PIC have a great diversity in population size and demographics. For example, the estimated population of Fiji is around 900,000, while Tuvalu and Nauru have an estimated population of approximately 11,000. This results in different market size, and potentially different needs of the Pacific Island Countries.<br>2. The PIC have a great variety in financial capacity, GDP per capita and their currency. The estimated GDP per capita in Nauru is around US$ 11,600, while Kiribati has an estimated GDP/capita of US$ 1,600. Also, there is a great diversity of currency on the PIC (e.g., New Zealand Dollar, US Dollar, Australian Dollar, Fiji Dollar, Samoan tālā, Tongan pa'anga, Solomon Islands Dollar), making the integration and coordination funds more complicated, as expressed under A10.<br>3. Great diversity in health outcomes, which might also result in different needs of health products, increasing the difficulty of product alignment, discussed under A8:	[2,3,24]

	a. Estimated life expectancy at birth in Kiribati is around 55, while Samoa has an estimated life expectancy of 76<br>b. Estimated infant mortality rate (deaths/per 1,000 live births of children <1 year) in Kiribati is around 41, while the estimated infant mortality rate in Fiji is around 12.5	
16. Shared cultural factors and values (e.g., language, traditions, etc.)	There is great diversity among Pacific Island Countries in culture, ethnicity and language. The PIC have traditionally been grouped by ethnogeographic and cultural factors into Melanesia, Micronesia and Polynesia. Some studies estimate that the Austronesian language family consists of more than 1200 languages, with hundreds of those languages still spoken in the Pacific Island Countries. These diversities in cultural factors and values might also influence the process of alignment on goals, purpose and operations of the pooled procurement mechanism and trust levels between PIC.	[1,22,36–38]
17. Existing political or structural mechanisms	1. The Pacific Islands Forum, which was established in 1971, is the region's leading political and economic policy organization. It consists of the PIC Heads of State.<br>Although many collaboration initiatives have come out of this Forum, some of which have been discussed under A14, there has been in a recent crisis in the Pacific Islands Forum. The Micronesian sub-group, which consists of Palau, Marshall Islands, the Federated States of Micronesia, Kiribati, and Nauru have quit the Pacific Island Forum over a dispute about the selection of the Forum's new director-general.<br>2. In 1981, the Government of Fiji set up the Fiji Pharmaceutical Services/Bulk Purchasing Scheme (FPS/BPS). The FPS/BPS provides medicines to Small Island States in the region, charging a 20% surcharge on the value of the order. The warehouse of the FPS/BPS has been provided by the Japan International Cooperation Agency (JICA). The estimated annual budget was less than AUS$ 0.5 million. The Scheme operates more as a supplier, rather than a pooled procurement mechanism. It has supplied Small Island States on an irregular basis. But since the orders consist often of very small quantities, it has not affected stock levels of the FPS/BPS significantly. There have been discussions on developing the FPS/BPS into an inter-country pooled procurement mechanism, but these plans have not been approved by other PIC, as discussed under A14.	[5,7,23,34]
**B. Pooled procurement organization**		
1. Organizational structure with clear roles and responsibilities	-	
2. Clear mandate	-	
3. Standardized and transparent procedures	-	
4. Sufficient, predictable and timely budget, either internal (through service fees) or external (through donors) to carry out pooled procurement	-	

5.	Sufficient, predictable and timely budget, either internal (through service fees) or external (through donors), to cover organizational expenses	-	
6.	Predictable, timely and efficient payment mechanism	-	
7.	Human resources (sufficient in numbers and expertise)	-	
8.	Sufficient technical capacity (e.g., procurement, quality assessment, forecasting, etc.)	-	
9.	Positive reputation	-	
10.	No conflict of interest	-	
11.	User-friendliness (both towards buyers and sellers)	-	
C.	**Suppliers**		
1.	Sufficient number of qualified suppliers	The lack of sufficient supplier incentives, discussed under C3, might result in reduced or no participation of reliable suppliers in the Pacific Island Countries. Unreliable suppliers, with lower quality standards or delivery performances, might jump in to fill up the gap in the pharmaceutical market, potentially resulting in poor quality medicines, shortages, wastage of financial resources, and poor health outcomes.<br>Currently, there are no regulatory authorities in place to assess supplier performance and qualifications.	[5]
2.	Sufficient production incentives	Production incentives are less relevant for PIC, because the PIC inter-country pooled procurement mechanism was aiming to procure high demand essential medicines that are frequently being consumed and produced. Therefore, supply incentives, outlined under C3, seem more relevant for PIC.<br>Production incentives are expected to be more relevant for disease specific, product specific or single source pooled procurement mechanisms, that aim to create a market for products that are not being produced, or not in sufficient quantities.	

3.	Sufficient supply incentives	Mainly due to its geographic location, market size and financial capacity, the Pacific Island Countries have had difficulty in providing sufficient supply incentives to suppliers:<br>- The remote and dispersed geographical location results in high distribution costs for suppliers;<br>- The already relatively low prices of medicines in the region make it unattractive for suppliers to supply the region;<br>- The small market size, both in terms of value and population, makes it unattractive for suppliers to bear the costs of distribution, as discussed under A9;<br>- The limited financial capacity of Pacific Island Countries results in the PIC not being able to pay for the costs of distribution themselves, as discussed under A3 and A10;<br>- There is no centralized payment mechanism or a shared currency that facilitates the prompt payment of suppliers, as partially discussed under A15;<br>- There is no centralized authority or secretariat in place that facilitates the procurement and/or distribution of medical products in the region;<br>- There is no centralized registration system, reducing the burden for suppliers to register health products in each Pacific Island Country individually, as discussed under A11.	[5]
4.	Sufficient number of distributors with favourable delivery terms	Based on experiences in the UNICEF Vaccine Independence Initiative (VII), further explained under A14, there have been multiple logistical difficulties in the PIC. Limited air travel services often result in doubling of the vaccines costs after shipping. This is partially caused by the volume of the shipment, since some shipments are only a handful of vials.<br>As discussed under A2, some experts have expressed concerns that the costs of freight might outweigh the benefits of the pooled procurement mechanism. Particularly, the countries and areas in the northern Pacific region (i.e., Palau, the Marshall Islands and the Federated States of Micronesia) are less likely to participate because of high transportation costs. They currently procure medicines mainly from Hawaii.	[15,27,39]

References

1. West FJ. Pacific Islands. In: Encyclopedia Britannica. Available from: https://www.britannica.com/place/Pacific-Islands
2. The Pacific Community (SPC). Pacific Islands 2020 Populations poster. 2020. Available from: https://spccfpstore1.blob.core.windows.net/digitallibrary-docs/files/1e/1e1bf203a79901a28e471a392a21d466.pdf?sv=2015-12-11&sr=b&sig=FPtk%2BwzlM7zx7ARzlkVsKr2Ss8W6EFIGDPxg6iuBy8M%3D&se=2021-08-31T11%3A56%3A19Z&sp=r&rscc=public%2C%20max-age%3D864000%2C%20max-stale%3D86400&rsct=application%2Fpdf&rscd=inline%3B%20filename%3D%22Pacific_Islands_2020_Populations_poster.pdf%22
3. The Pacific Community (SPC). Pocket Statistical Summary 2020. 2020. Available from: https://spccfpstore1.blob.core.windows.net/digitallibrary-docs/files/30/30a079939bb8b7deca412cae5a96a0f5.pdf?sv=2015-12-11&sr=b&sig=vimTJxhInGQ0tJpG%2FLKq2wXFxjmQ1jQFNNDZjUMy6ho%3D&se=2021-08-31T11%3A59%3A48Z&sp=r&rscc=public%2C%20max-age%3D864000%2C%20max-stale%3D86400&rsct=application%2Fpdf&rscd=inline%3B%20filename%3D%22Pocket_statistical_Summary_20.pdf%22
4. The World Bank. Current health expenditure (% of GDP) - Fiji, Papua New Guinea, Solomon Islands, Vanuatu, Micronesia, Fed. Sts., Kiribati, Marshall Islands, Nauru, Palau, American Samoa, French Polynesia, Samoa, Tonga, Tuvalu | Data. Available from: https://data-worldbank-org.eur.idm.oclc.org/indicator/SH.XPD.CHEX.GD.ZS?locations=FJ-PG-SB-VU-FM-KI-MH-NR-PW-AS-PF-WS-TO-TV
5. Arifaj-Blumi D. Feasibility Study on Pooled Procurement for Pacific Island Countries. Suva, Fiji: World Health Organization for the Western Pacific Region; 2007. Report No.: (WP)PHA/PIC/EDM/3.2/01.
6. Shibuya E. The problems and potential of the Pacific Islands forum. In: The Asia-Pacific: A region in transition. Asia-Pacific Center for Security Studies; 2004. p. 102–15.
7. Carreon B, Doherty B. Pacific Islands Forum in crisis as one-third of member nations quit. The Guardian. 2021 Feb 8; Available from: http://www.theguardian.com/world/2021/feb/09/pacific-islands-forum-in-crisis-as-one-third-of-member-nations-quit
8. World Health Organization Regional Office for the Western Pacific, South Pacific Bureau for Economic Cooperation. WHO/SPEC Meeting on Technical Cooperation Among South Pacific Countries/Areas in Pharmaceutical Supplies. Suva, Fiji; 1978 Dec. Report No.: ICP/DPM/002. Available from: https://apps.who.int/iris/bitstream/handle/10665/207209/ICP_DPM_002_19781208_eng.pdf?sequence=1&isAllowed=y
9. World Health Organization. Thirty-second World Health Assembly. Geneva, Switzerland: World Health Organization; 1979 May. Available from: https://apps.who.int/iris/bitstream/handle/10665/153658/WHA32_1979-REC-1_eng.pdf?sequence=1&isAllowed=y
10. World Health Organization Regional Office for the Western Pacific, South Pacific Bureau for Economic Cooperation, United Nations Development Fund. Working Group on Techical Cooperation in Pharmaceutical Supplies in the South Pacific. Auckland, New Zealand; 1979 Aug. Available from: https://apps.who.int/iris/bitstream/handle/10665/207112/19790830_NEZ_eng.pdf?sequen

ce=1&isAllowed=y
11. South Pacific Bureau for Economic Cooperation. Director's Annual Report 1979/1980. Suva, Fiji; 1980 May. Report No.: SPEC (80) 8.
12. World Health Organization Regional Office for the Western Pacific. The Work of WHO in the Western Pacific Region, 1 July 1981 - 30 June 1983. Manila, Philippines: World Health Organization Regional Office for the Western Pacific; 1983 Jun. (Biennial report of the Regional Director to the Regional Committee for the Western Pacific). Report No.: WPR/RC34/3.
13. World Health Organization Regional Committee for the Western Pacific. Drug Supply Management in the South Pacific. Manila, Philippines: World Health Organization; 1987 Jul. Report No.: WPR/RC38/11. Available from: https://apps.who.int/iris/bitstream/handle/10665/141429/WPR_RC038_11_Drug_Supply_1987_en.pdf?sequence=1&isAllowed=y
14. South Pacific Bureau for Economic Cooperation. Director's Annual Report 1980/1981. Suva, Fiji; 1981 Jun. Report No.: SPEC (81) 6. Available from: http://www.forumsec.org/wp-content/uploads/2017/10/1980-1981.pdf
15. Mendoza OM. Regional Pooled Procurement of Essential Medicines in the Western Pacific Region: An Asset or a Liability? The University of Auckland: Centre for Development Studies; 2010. Available from: https://cdn.auckland.ac.nz/assets/arts/Departments/development-studies/documents/working-paper-series/O'neal%20Menoza%20WPS%201_20102.pdf
16. World Health Organization Regional Office for the Western Pacific. Informal Consultation on Pooled Procurement of Essential Medicines for Pacific Island Countries, Nuku'alofa, Tonga, 6 August 2007. Nuku'alofa, Tonga: WHO Regional Office for the Western Pacific; 2007 Aug. Report No.: RA/2007/GE/44(TON). Available from: http://iris.wpro.who.int/handle/10665.1/10698
17. World Health Organization Regional Office for the Western Pacific. Yanuca Island Declaration. Manila, Philippines; 1995 Mar. Report No.: WHO/HRH/95.4. Available from: https://www.pihoa.org/wp-content/uploads/2019/08/Yanuca-Island-Declaration.pdf
18. World Health Organization Regional Office for the Western Pacific. Meeting of the Ministers of Health for the Pacific Island countries. Rarotonga, Cook Islands: Manila : WHO Regional Office for the Western Pacific; 1997 Aug. Report No.: RS/97/GEI19(COK). Available from: http://iris.wpro.who.int/handle/10665.1/5951
19. World Health Organization Regional Office for the Western Pacific. The Palau Action Statement on Healthy Islands. Koror, Republic of Palau: Manila: WHO Regional Office for the Western Pacific; 1999 Mar. Report No.: WPR/HRH/DHI/99.1. Available from: http://iris.wpro.who.int/handle/10665.1/5565
20. World Health Organization Regional Office for the Western Pacific. Workshop on Pharmaceutical Policies and Access to Good Quality Essential Medicines for Pacific Island Countries, Suva, Fiji, 30 August - 1 September 2006. Manila: WHO Regional Office for the Western Pacific; 2006. Available from: http://iris.wpro.who.int/handle/10665.1/6211
21. World Health Organization Regional Office for the Western Pacific. Informal Consultation on Pooled Procurement of Pharmaceuticals for Pacific Island Countries, Suva, Fiji, 29-30 March 2007. Suva, Fiji: WHO Regional Office for the Western Pacific; 2007 Mar. Report No.: RSI2007/GEl43IF1J. Available from: http://iris.wpro.who.int/handle/10665.1/6248
22. World Health Organization Regional Office for the Western Pacific. Eighth Meeting of

Ministers of Health for the Pacific Island Countries, Madang, Papua Guinea, 7-9 July 2009. Manila : WHO Regional Office for the Western Pacific; 2009. Report No.: RS/2009/GE/43(PNG). Available from: https://apps.who.int/iris/handle/10665/207152
23. Dornan M, Newton Cain T. Regional Service Delivery among Pacific Island Countries: An Assessment: Pacific regionalism. Asia Pacific Policy Stud. 2014 Sep;1(3):541–60.
24. UNICEF Pacific Office. Situation Analysis of Children in the Pacific Island Countries. Suva, Fiji; 2017 Dec. Available from: https://www.unicef.org/pacificislands/media/661/file/Situation-Analysis-Pacific-Island-Countries.pdf
25. The World Bank. Pacific Possible: Long-term Economic Opportunities and Challenges for Pacific Island Countries. 2017 Aug. Report No.: ACS22308. Available from: http://documents1.worldbank.org/curated/en/168951503668157320/pdf/ACS22308-PUBLIC-P154324-ADD-SERIES-PPFullReportFINALscreen.pdf
26. The World Bank. Working Together - Revised Pacific Possible Background Note. 2016. Available from: http://doi.wiley.com/10.1002/app5.45
27. World Health Organization Regional Office for the Western Pacific. Workshop on Pharmaceutical Policies, Intellectual Property Rights and Access to Essential Medicines for Pacific Island Countries, Nuku'alofa, Tonga, 7-9 August 2007. Nuku'alofa, Tonga: WHO Regional Office for the Western Pacific; 2007 Aug. Report No.: RS/2007 /GE/29(TON). Available from: https://apps.who.int/iris/bitstream/handle/10665/208641/RS_2007_GE_29_TON_eng.pdf?sequence=1&isAllowed=y
28. World Health Organization Regional Office for the Western Pacific. Pacific Island Meeting on Subregional Regulatory Systems for Medicine, Suva, Fiji, 28 February - 1 March 2019. Suva, Fiji: Manila: WHO Regional Office for the Western Pacific; 2019 Jul. Report No.: RS/2019/GE/10(FJI). Available from: https://apps.who.int/iris/handle/10665/326081
29. World Health Organization Regional Office for the Western Pacific. Western Pacific Regional Action Agenda on Regulatory Strengthening, Convergence and Cooperation for Medicines and the Health Workforce. Manila: WHO Regional Office for the Western Pacific; 2020. Available from: http://iris.wpro.who.int/handle/10665.1/14516
30. World Health Organization Regional Office for the Western Pacific. Regional Framework for Action on Access to Essential Medicines in the Western Pacific (2011-2016). Manila: WHO Regional Office for the Western Pacific; 2012. Available from: https://apps.who.int/iris/handle/10665/207519
31. Macé C. Pooled Procurement of Insulin and Associated Supplies. Health Action International; 2022. Available from: https://haiweb.org/wp-content/uploads/2022/02/Pooled_Procurement_of_Insulin.pdf
32. Gani A. Health care financing and health outcomes in Pacific Island countries. Health Policy Plan. 2009 Jan 1;24(1):72–81.
33. World Health Organization Regional Office for the Western Pacific. Assistive Technology Procurement Study. Manila: WHO Regional Office for the Western Pacific; 2020. Available from: https://apps.who.int/iris/handle/10665/334368
34. World Health Organization. Regional workshop on strengthening quantification and procurement of essential medicines. Report of the workshop held in New Delhi, India, 10-12 June 2014. 2014; Available from: https://apps.who.int/iris/handle/10665/206213
35. Nemzoff C, Chalkidou K, Over M. Aggregating Demand for Pharmaceuticals is Appealing, but Pooling Is Not a Panacea. Center for Global Development; 2019 May.

Available from: https://www.cgdev.org/sites/default/files/aggregating-demand-pharmaceuticals-appealing-pooling-not-panacea.pdf
36. Asian Development Bank. Toward a New Pacific Regionalism. Asian Development Bank; 2005 Jan. Report No.: 081105. Available from: https://www.adb.org/publications/toward-new-pacific-regionalism
37. Gray RD, Drummond AJ, Greenhill SJ. Language Phylogenies Reveal Expansion Pulses and Pauses in Pacific Settlement. Science. 2009 Jan 23;323(5913):479–83.
38. Costa M, Sharp R. The Pacific Island Countries Fiji, Papua New Guinea (PNG), Samoa, Solomon Islands, Vanuatu and Tuvalu. 2011. Available from: https://www.unisa.edu.au/siteassets/episerver-6-files/documents/eass/hri/gender-budgets/pic.pdf
39. The World Bank, GAVI Alliance. Brief 13: UNICEF – Vaccine Independence Initiative (VII). 2010. Available from: https://www.who.int/immunization/programmes_systems/financing/analyses/Brief_13_Vaccine_Independence_Initiative.pdf
