# Supplementary material for: Does structural form matter? A comparative analysis of pooled procurement mechanisms for health commodities
Source: Global Health. 2023 Nov 23;19:90. doi: 10.1186/s12992-023-00974-1 (PMC10668364; doi:10.1186/s12992-023-00974-1)
Supplement: Supplementary file 3 — Additional file 3. [file 12992_2023_974_MOESM3_ESM.pdf]

Mature Stage – Global Drug Facility (GDF)

General characteristics and historical developments

Tuberculosis (TB) is a bacterial disease that is caused by *Mycobacterium tuberculosis*. TB is a communicable disease that is spread through air, and can lead to death if left untreated. Currently, TB is estimated to cause 1.6 million deaths worldwide per year [1].

A rich history of developments in the fight against TB preceded the creation of the Global Drug Facility (GDF). Before anti-tuberculosis medicines existed, treatment was mainly based on rest and diet. Then, in the 1950s, long-course treatment, which consists of a combination of isonicotinic acid hydrazide (INH), streptomycin (SM) and para-aminosalicylic acid (PAS) became available. Although this "triple therapy" regimen was effective, it required 24 months of continuous treatment [2,3]. In the 1970s, with the introduction of rifampicin in the anti-TB drug regimen, the treatment period was reduced to 6 to 8 months. This was referred to as the short-course treatment [2].

An important breakthrough came with the introduction of the DOTS Strategy (*Directly observed treatment, short-course*), pioneered by Dr Karel Styblo. This strategy consisted of an integrated approach consisting of 5 elements [3]:

1. Political commitment to effective treatment;
2. An assured supply of medications;
3. Diagnosis by sputum microscopy;
4. Analysis of the entire cohort of patients initiated on treatment;
5. Directly-observed treatment, especially during the first 2 months

In 1988, the World Health Organization (WHO) created a TB Unit, called the Stop TB Department, within its Communicable Diseases Programme [4]. Three years later, in 1991, the World Health Assembly (WHA44.8) set an ambitious target for TB control of diagnosing 70% of cases and curing 85% of sputum-positive patients by the year 2000 [4]. In 1993, the World Health Organization (WHO) went a step further, and declared TB a global emergency [2]. This was a significant step in increasing awareness, it put the fight against TB on high up the global health agenda.

In 1998, a WHO Ad Hoc Committee on Tuberculosis was formed in London to discuss the ongoing efforts to reach the goals set up during WHA44.8 by 2000 and come up with potential solutions. During this meeting, the Committee identified a reliable medicine supply as one of the key limiting factors, and suggested the establishment of a Global Drug Facility [5–7]. This facility would be responsible for procurement and distribution of TB medicines by providing a revolving fund and procurement mechanism; an effective mechanism between donors and recipient countries; international technical resource that assists strengthening of recipient-country procurement capacities; and monitor and report the prices of TB medicines [7].

Following this meeting, a reorganization took place at the WHO. The Global TB Programme was closed to integrate the managerial functions of WHO's separate control programs [5]. Around the same time, The Stop TB Initiative, which was the predecessor of the Stop TB Partnership, was founded in November 1998 under WHO's umbrella [5,8].

In March 2000, another milestone meeting was organized by the Stop TB Initiative in Amsterdam, the Ministerial Conference on TB and Sustainable Development. This meeting produced the Amsterdam Declaration [4,6]. Later that year, The World Health Assembly (WHA53.1) endorsed the establishment of the Stop TB Initiative [9].

In February 2001, the interim Coordinating Board devised a structure for the Partnership. This seems like one of the steps that transformed the Stop TB Initiative into the Stop TB Partnership.[4] One month later, the Global Drug Facility was launched on 24 March 2001, with the goal to increase access to high quality TB medicine [6].

Another crucial development was the establishment of the Global Fund to Fight AIDS, Tuberculosis and Malaria in 2002 as a new and autonomous global funding mechanism after the G8 endorsed its founding a year earlier in Genoa [10]. In 2006, UNITAID was founded with the goal to scale up access to treatment of major diseases in low- and middle-income countries, with an emphasis on tuberculosis, malaria, and HIV/AIDS [11,12].

In the following years, the GDF has achieved many positive outcomes in terms of market shaping, price reduction, technical assistance and quality assurance. Also, its operations have evolved over time in response to changes in epidemiology, the funding landscape, country needs, product availability and other factors, including those changes generated by the GDF itself. Most of these achievements and organizational developments are described in the Table below.

In 2018, the United Nations encouraged the use of the Global Drug Facility in a UN Resolution (A/RES/73/3) [13].

A timeline of important events in the fight against TB are shown in Figure 1.

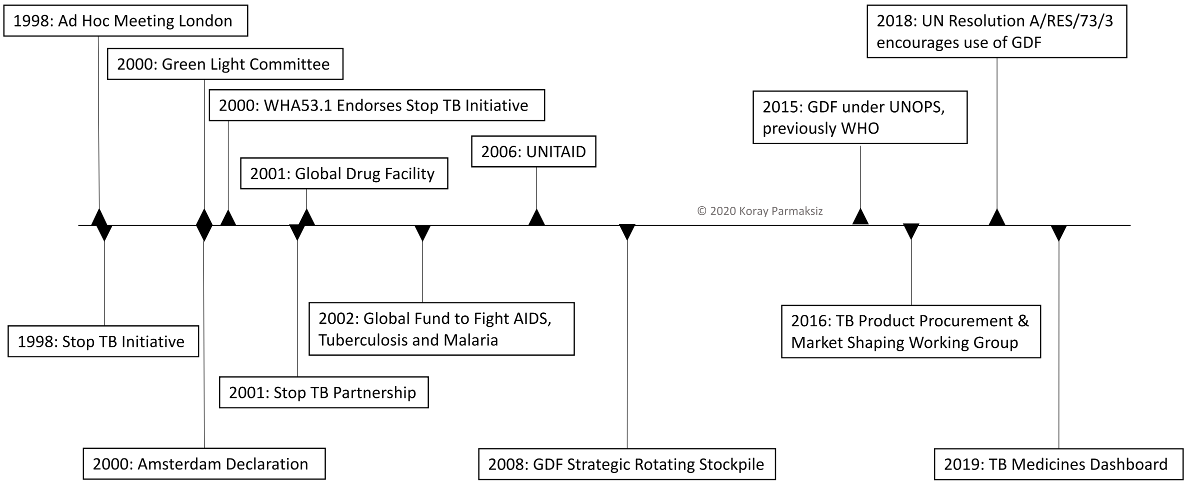

Figure 1. Timeline of events in the fight against TB.

Essential elements

We have identified several essential elements that have contributed to the successful implementation and operations of the Global Drug Facility's pooled procurement mechanism.

1. **Tuberculosis was perceived as an urgent problem by buyer countries and the global health arena**

The combination of three main factors created the preconditions to raise global attention: the nature of the disease, the high disease burden, and the possibility to cure TB with the right treatment. As mentioned above, TB is an airborne communicable disease caused by the *Mycobacterium tuberculosis*. The bacteria are transmittable from human-to-human and can lead to death if left untreated [2,14]. TB has a high global disease burden. The World Health Organization (WHO) estimates that a quarter of the world population has a TB infection, with 30 high burden TB countries, mainly low- and middle-income countries, accounting for 90% of the TB cases [14]. Currently, TB is estimated to cause 1.6 million deaths per year [1]. The development of an effective multi-component treatment strategy, called DOTS (*Directly observed treatment, short-course*), was an important breakthrough for curing people with TB [2]. However, many high burden TB countries experienced challenges in implementing the DOTS strategy. These challenges included lack of financial resources to procure TB medicines, lack of access to high quality TB medicines, inefficient procurement systems, lack of national TB programs, a small market size for TB medicines and diagnostics, and limited human resources to accurately diagnose and treat TB [6,11,15].

In 1993, TB climbed its way up on the global health agenda after the WHO declared TB a global health emergency, acknowledging that the spread of TB could only be contained if a universal approach would be taken [16]. This global attention brought many key actors together, including global health organizations, high burden TB countries, donors and NGOs. One important initiative that emerged from this multi-actor engagement in the battle against TB was the Stop TB Partnership [4,6]. This partnership led to the establishment of the Global Drug Facility (GDF). The GDF was set up to solve the problems of lacking high quality and affordable TB medicines, which was seen as an important barrier in the rapid expansion of the DOTS strategy [6,7,17].

Increased global awareness, the availability of external funding, and the multi-stakeholder approach put TB also on the national agenda of potential buyers. These buyers realized that pooled procurement, as organized by the GDF, could provide a solution to the problems they experienced in the fight against TB.

2. **Sufficient, predictable and timely budget, either internal or external (through donors)**

The nature of TB, which spreads from human-to-human through air poses a potential health threat for higher income countries. This threat, in combination with the high disease burden and the fact that TB is curable with the right treatment, allowed the "TB-sector" to attract a significant amount of external funding from donor organizations and development aid from high-income country foreign ministries. During early years, The GDF provided grants to buyer countries with funding from external donors, including the Global Fund, USAID, CIDA, The Government of the Netherlands, the World Bank, DFID and UNITAID. In recent years, the GDF has shifted from grant provision to direct procurement, which forces buyer countries to procure medicines and diagnostics through the GDF with government health budgets or external funding, mainly provided by the Global Fund to Fight AIDS, Tuberculosis and Malaria (Global Fund). In 2020, 54 countries procured through the GDF using domestic funding.

To cover organizational expenses, the GDF does not charge a service fee for orders that are placed through its direct procurement services. Instead, the GDF's operational expenses are covered through donor funding. In 2011, GDF mentioned that operational expenses, including "human resources, advocacy, quality management, general operating costs, meetings/workshops, travel, expert advice and missions/desk audits" accounted for US$ 6 million per year. Currently, the operational costs of GDF are largely covered by USAID, which are covered through congressional

budget. According to the US Congressional Budget Justification (Fiscal Year 2021), USAID's total funding to the GDF was US$ 15 million in 2019 [18,19].

3. Organizational structure with clear roles and responsibilities

The GDF is run by a dedicated secretariat with sufficient human resource capacity. According to the Stop TB Partnership website, the GDF secretariat consists of 41 staff members, of which around 30 are based at the headquarters in Geneva, Switzerland; and the remaining staff operating externally in different regions, mainly in high burden TB countries. The staff of the GDF are highly trained and highly specialised, focusing on many areas, including TB advocacy, market shaping, sourcing, stakeholder alignment and coordination, demand forecasting and quantification, technical assistance and capacity building, tendering, contract management with suppliers, oversight of quality assurance, warehousing, distribution, and data management [20]. GDF's organizational structure with a dedicated secretariat, sufficient in numbers and expertise, allows the GDF to provide a rounded procurement service.
In addition, the GDF secretariat is surrounded by many global health and technical organizations that are part of the Stop TB Partnership, with whom they collaborate closely.
Another strength of GDF's operations is that the GDF has outsourced certain tasks to reliable external parties that can carry out the procurement and related services more efficiently. For example, the GDF has outsourced the task of procurement and warehousing to procurement agents:

- First-Line Drugs (FLDs): IAPSO/UNDP (2001-2006), GTZ (2006-2009), PFSCM (2009-2010), GTZ (2010-2012), IDA Foundation (2012-present)
- Second-Line Drugs (SLDs): IDA Foundation (2007 – present)
- Diagnostics: GTZ (2006-2012), GIZ (2012-2015), GDF in-house (2015 – present)

Other examples of outsourced services of the GDF are quality assessment, which have been outsourced to a quality-control agent named Société Générale de Surveillance (SGS), Nederland BV, and distribution, which have been outsourced to freight forwarders.
In addition, the GDF requires that all finished pharmaceutical products are either WHO-prequalified or approved by a Stringent Regulatory Authority (SRA). When there are no products available with WHO-prequalification or SRA-approval, an External Review Panel (ERP) reviews the product and might give a recommendation to procure the product for up to 12 months [21].

4. Positive reputation and a rounded procurement service

The GDF does not merely operate as an intermediate organization that consolidates demand and carries out pooled procurement. It provides a rounded procurement service to incentivize buyers to participate in the mechanism. For example, the GDF provided grants to eligible buyer countries at initiation [6]. Currently, a Flexible Procurement Fund (FPF) provides financial flexibility to buyers that have difficulty to adhere to GDF's prepayment conditions [22]. The GDF also supports buyer countries with capacity building and technical assistance in several areas, including demand planning and stock monitoring [11,23,24]. The GDF and the Stop TB Partnership, under which the GDF is housed, both operate under the United Nations umbrella. This institutional backing legitimized GDF's operations and provided the GDF high-level access to high burden TB countries. Reciprocally, the GDF involves representatives of high burden TB countries in its governance mechanism as board members of the Stop TB Partnership [4,25,26]. This governance structure increased the buyer's trust in the GDF. GDF's user-friendly services with high client satisfaction ratings [15], combined with a positive track record of increasing access to quality-assured and affordable TB medicines have reinforced the GDF's positive reputation to attract and incentivize potential buyers to procure through its pooled procurement organization.

5. Supplier incentives

Providing sufficient production incentives and shaping the market was more relevant for GDF's pooled procurement mechanism. Prior to GDF, there was a lack of quality generic TB medicines [6,11]. The GDF has taken multiple market shaping approaches and provided several production

incentives to suppliers for the production of quality TB medicines. As part of its market shaping efforts, the GDF accelerated the simplification and standardization of complex TB treatment regimens by consolidating demand in buyer countries around affordable fixed-dose combination (FDC) treatments and incentivizing its production [27]. Similarly, the GDF has played a critical role in driving research and development, procurement and adoption of paediatric TB medicines [15,28].
The GDF also provided many production incentives to suppliers. For example, the TB medicines market, which was too small and unpredictable, made it risky and expensive for suppliers to carry stock. The GDF tackled this by establishing a Strategic Rotating Stockpile (SRS). The SRS created a buffer stock and levelled off the erratic demand of buyers, resulting in sharing the risk of stock carrying with suppliers and reduced delivery lead times of TB medicines [29]. The GDF also provided suppliers long-term framework agreements giving suppliers a certain degree of security to produce, as long as they adhered to the agreed conditions and quality standards [30]. Also, the quality of mainly domestically produced TB medicines was improved in close collaboration with the WHO Prequalification Programme. The GDF achieved annual fee exemption of TB medicines with relatively low profit margins from the WHO Prequalification program, lowering the barrier for manufacturers to obtain prequalification for their products and making the production of prequalified TB products economically feasible [29]. This fee-exemption and GDF's quality-assurance policy requirements forced suppliers to adhere to either WHO-prequalification or Stringent Regulatory Authority standards if suppliers wanted to get access to a consolidated TB medicines market provided by the GDF [21].
In addition to production incentives, the GDF also provides suppliers with several supply incentives, such as the adoption of a predictable, timely and single currency payment mechanism to facilitate prompt payment of suppliers and the packaging of TB medicines in 4 languages by the GDF to reduce the supplier's burden of repackaging and translating [24,31].

Essential elements/actor	*Global Drug Facility (GDF)*	References
**A. Buyers**		
**All buyers <u>need</u> to have individually**:		
1. Perceived problem for which pooled procurement may be a solution (problem)	Several problems have been mentioned in widespread adoption of the DOTS treatment and reaching the targets set up during WHA44.8, including lack of financial resources for TB medicines, lack of access to quality TB medicines, inefficient procurement systems, lack of national TB programs, lack of standardized treatment regimens, small market size, and limited human resources.	[6,11,15,32]
2. Motivations that outweigh the opportunity costs	The motivations for buyer countries to procure through the GDF outweigh the opportunity costs. The GDF provides a rounded procurement service to buyer countries, including:<br>- As explained under A3, the GDF provided grants to eligible buyer countries previously. Currently, eligible countries can procure through GDF with Global Fund funding, or other sources of donor funding;<br>- Access to quality-assured and affordable TB medicines from prequalified suppliers;<br>- Capacity building and technical assistance in several areas, including demand planning;<br>- As explained under B11, the GDF provides a user-friendly platform that minimizes the risks involved with procurement for buyers;<br>- As further explained under B1, the high burden TB countries are partially involved in the decision-making of the GDF as Board members of the Stop TB Partnership.	[6,33]
3. Budget, either internal or external (through donors)	Prior to the WHO Ad Hoc Meeting in London in 1998 on the ongoing efforts to reach the goals set up in WHA44.8, a report was written to assess the status of TB in 22 high burden countries. The report pointed out that many of the high TB burden countries lack sustainable financial resources to procure quality TB medicines. The lack of financial resources of buyer countries was also seen during the first 2 years of the GDF's operations, where 65 countries applied to the GDF for grants for TB medicine, of which 46 got approved. The reasons for application included: "lack of finances to buy drugs due to financial crises, lack of foreign currency to procure drugs, inefficient or slow procurement mechanisms, drug quality concerns, increased demand for drugs due to the HIV/ AIDS epidemics, deteriorating health structures, socio-economic changes, war, loss of donor support, etc." GDF provided grants to countries with a Gross National Product (GNP) <US$3000, with a particular focus on countries with a GNP <US$1000, usually for a duration of 3 years. At initiation, the source of GDF's grants mainly came from four core donors: The Canadian International Development Agency (CIDA), The Government of the Netherlands, The United States Agency for International Development (USAID), and the World Bank. In the following years, the UK Department for International Development (DFID) and UNITAID also contributed significant funding to GDF.<br>In recent years, the GDF has shifted from grant provision to direct procurement, which forces buyer countries to procure medicines and diagnostics through the GDF with government health budgets or external funding,	[4,6,7,11,15,18,32,34–36]

		mainly provided by the Global Fund to Fight AIDS, Tuberculosis and Malaria (Global Fund). The value of direct procurement increased from US$ 19 million in 2007 to US$ 103 million in 2011. The Global Fund obliges countries that receive Global Fund funding to procure through the GDF for Second-Line Drugs (SLD), while this is not obligatory for First-Line Drugs and diagnostics. In 2020, 86 countries procured with Global Fund funding. The countries that procured through the GDF with domestic funding increased from 12 in 2009 to 54 In 2020.	
4.	Sufficient technical capacity (e.g., demand forecasting)	As explained under A3, many countries out of the 65 countries applied to grants for TB medicine, applied due to a lack of financial and technical capacity to procure quality and affordable medicine. Lacking technical capacity included inefficient or slow procurement mechanisms and inaccurate demand forecasts.<br>Buyers also experienced technical capacity constraints with timely and efficient product registration, and in the sufficiency of qualified human resources	[6,7,15]
5.	Compatible laws, regulations and policies that allow for (international) pooled procurement	Little public information available. However, based on expert opinion we understand that some buyer countries experience challenges with national regulations that only allow for domestic procurement, instead of procurement from international mechanism such as the GDF. Other restricting regulations are around payment of international procurement mechanisms, restricting advance payments.<br>Another example of regulatory barriers was the lack of a fast-track registration or waiver system in buyer countries resulting in delays of importing the health products into the buyer country.	[37,38]
**If buyer's mechanism, all buyers combined, <u>need</u> to have:**			
6.	Demonstrated willingness to solve their problem collectively through pooled procurement (shared vision)		
7.	Alignment on goals, purpose and operations of the pooled procurement mechanism (shared plan)		
8.	Joint need for specific products (product alignment)		
9.	Sufficient market size		
10.	Sufficient and stable financial capacity		
11.	Regulatory harmonization (e.g., shared quality standards, joint assessment, market authorization, etc.)		
12.	Trust (in other buyers and the pooled procurement organization)		

13. Transparent data and information sharing
14. No history of conflict or failed collaboration
15. Homogeneity of buyer's characteristics related to their needs
16. Shared cultural factors and values (e.g., language, traditions, etc.)
17. Existing political or structural mechanisms

B. Pooled procurement organization

1. Organizational structure with clear roles and responsibilities	The Global Drug Facility (GDF) is operating under the Stop TB Partnership. Until 2015, the Stop TB Partnership and the GDF were housed under the World Health Organization (WHO). Since 2015, both the Stop TB Partnerships and the GDF are housed under UNOPS.<br>The GDF has a dedicated Secretariat based in Geneva, Switzerland with clear roles and responsibilities. As further explained under B7, among the GDF secretariat's task are providing administrative support, manages procurement and related services (e.g., market shaping, sourcing, demand planning) and provide technical assistance.<br>The governance structure of the GDF, where roles and responsibilities are divided between the Stop TB Partnership, the United Nations Office for Project Services (UNOPS), and the GDF Secretariat allows the partners to focus on their specific task, hold each other accountable, and strengthen GDF's operations.<br>The Stop TB Partnership mainly provides funding and technical assistance through partners to the GDF and buyer countries. Operating under the United Nations umbrella, first under the WHO, and now under UNOPS, provides the GDF high-level access to high burden TB countries. In turn, the Board of the Stop TB Partnership consists of many high burden TB country representatives, as well as global health organization, international donors and technical agencies. This structure, where a wide variety of stakeholders are actively part of GDF's operations, contributes to legitimizing GDF's operations in the buyer countries, as well as the global health arena.	[4,6,26,33]

2.	Clear mandate	At initiation, GDF had a clear and focused mandate:<br>- To secure sustainable access to quality-assured TB medicine to facilitate expansion of the DOTS treatment;<br>- To catalyse rapid DOTS expansion to achieve the WHO targets set at WHA44.8;<br>- To stimulate global political and public support for public funding of TB medicines;<br>- To secure global TB control and eventually eliminate TB.<br><br>This mandate was provided by a wide variety of stakeholders, including the WHO, United Nations, World Bank, USAID, CIDA (Canadian International Development Agency), Stop TB Partnership, high TB burden countries, such as India and Uganda, foundations (e.g., Royal Netherlands Tuberculosis Association, Rockefeller Foundation). As explained under B1, the wide variety of stakeholders currently involved in GDF's operations allows the GDF's mandate to be widely acknowledged.	[6,11,17]
3.	Standardized and transparent procedures	The GDF has set up several standardized and transparent procedures accessible on their website regarding its procurement processes. These procedures include procurement request forms for buyers, quality assurance policy documents, product catalogues, indicative prices of products for budgeting purposes, adverse drug reaction forms, and a procurement and planning delivery document with indicative lead times for products. In addition, GDF's procurement agent, IDA Foundation, publishes documents for suppliers, such as invitations to bid and packaging guidelines.	[30,39–44]
4.	Sufficient, predictable and timely budget, either internal (through service fees) or external (through donors) to carry out pooled procurement	As explained under A3, the GDF provided grants to buyer countries with funding from external donors, including the Global Fund, USAID, CIDA, The Government of the Netherlands, the World Bank, DFID and UNITAID.<br>Recently, the GDF has transitioned from grant provisions to direct procurement, where buyer countries procure TB-related health products with domestic funding or donor funding, which is mainly provided by the Global Fund. In 2020, 54 countries procured through the GDF using domestic funding.<br>In 2012, 73% of GDF's funding for FLDs, which amounted to US$ 32.5 million, came from the Global Fund. This percentage was 89% for SLDs, corresponding to US$ 57.8 million.	[6,45]
5.	Sufficient, predictable and timely budget, either internal (through service fees) or external (through donors), to cover organizational expenses	The GDF does not charge a service fee for orders that are placed through its direct procurement services. Instead, the GDF's operational expenses are covered through donor funding. In 2011, GDF mentioned that operational expenses, including "human resources, advocacy, quality management, general operating costs, meetings/workshops, travel, expert advice and missions/desk audits" accounted for US$ 6 million per year. Currently, the operational costs of GDF are largely funded by USAID, which are covered through congressional budget. According to the US Congressional Budget Justification (Fiscal Year 2021), USAID's total funding to the GDF was US$ 15 million in 2019.	[6,18,18,19,36]

6. Predictable, timely and efficient payment mechanism	In general, buyers are required to pay the GDF before receiving the shipment. After placing the order, the buyer receives an invoice including all the related costs of the order. Then, after the buyer accepts the invoice, the order will be finalized by signing the contract. The payment takes place in the form of 'advance payment', which obliges the buyer to transfer the money to the procurement agent within 30 days after signature of the contract, or the buyer provides a 'bank guarantee' issued by a reputable bank to the procurement agent within 30 days after signature of the contract. All payments are done in US$, with the buyer being responsible for the risk of currency fluctuations.<br><br>In 2014, however, the GDF set up a Flexible Procurement Fund (FPF) with funding from USAID, to offer financial flexibility to buyers that have difficulty to adhere to GDF's prepayment conditions. With the FPF, GDF allows buyers to pay within 30 days after receiving the order. Until 2019, 16 countries had made use of the FPF.	[22,24,31]
7. Human resources (sufficient in numbers and expertise)	According to the Stop TB Partnership website, the GDF secretariat consists of 41 staff members. Of these, around 30 are operating from Geneva, Switzerland; with the remaining staff operating externally in different regions, mainly in high burden TB countries.<br>The staff of the GDF are highly trained and highly specialised, focusing on many areas, including advocacy, market shaping, stakeholder alignment and coordination, demand forecasting and quantification, technical assistance, capacity building, supply, sourcing, tendering, contract management with suppliers, oversight of quality assurance, warehousing, distribution, and data management.	[20]
8. Sufficient technical capacity (e.g., procurement, quality assessment, forecasting, etc.)	As explained under B1 and B7, the GDF has sufficient technical capacity to carry out procurement and related services, with human resources that have sufficient expertise and experience. In addition, the GDF secretariat is surrounded by many global health and technical organizations that are part of the Stop TB Partnership, with whom they collaborate closely.<br><br>Another strength of GDF's operations is that the GDF has outsourced certain task to reliable external parties that can carry out the procurement and related services more efficiently.<br>For example, the GDF has outsourced the task of procurement and warehousing to procurement agents:<br>- First-Line Drugs (FLDs): IAPSO/UNDP (2001-2006), GTZ (2006-2009), PFSCM (2009-2010), GTZ (2010-2012), IDA Foundation (2012-present)<br>- Second-Line Drugs (SLDs): IDA Foundation (2007 – present)<br>- Diagnostics: GTZ (2006-2012), GIZ (2012-2015), GDF in-house (2015 – present)<br><br>Other examples of outsourced services of the GDF are quality assessment, which have been outsourced to a quality-control agent named The Société Générale de Surveillance (SGS), Nederland BV, and distribution, which have been outsourced to freight forwarders.	[6,21]

	In addition, the GDF requires that all finished pharmaceutical products are either WHO-prequalified or approved by a Stringent Regulatory Authority (SRA). When there are no products available with WHO-prequalification or SRA-approval, an External Review Panel (ERP) reviews the product and might give a recommendation to procure the product for up to 12 months.	
9. Positive reputation	The GDF provides a rounded procurement service to its buyers that covers a wide variety of areas, which have been explained under B7. Specific examples of these services towards buyers include:<br>- Capacity building of demand planning and monitoring through the QuanTB tool that allows for more insight in the supply situation of the country and an early alert system for shortages or stock-outs. This can be used for more accurate and timely demand forecasting;<br>- As explained under B7, a Flexible Procurement Fund (FPF) that provides financial flexibility to buyers that have difficulty to adhere to GDF's prepayment conditions;<br>- As explained under B11, the GDF provides user-friendly services with high client satisfaction ratings, which reinforces the positive reputation of the GDF;<br>- GDF's achievements in reducing prices have also reinforced its positive reputation. For example, it has managed to reduce prices of SLDs by 60% between 2012 and 2019,<br><br>Specific examples of services that contribute to a positive reputation towards suppliers, such as the SRS warehouse, are mentioned under C2 and C3.<br><br>The GDF has also managed to establish a positive reputation in the global health arena. Its operations and pooled procurement mechanism have served as a model for other disease-specific pooled procurement mechanism (e.g., Asthma Drug Facility, UNITAID, Global Fund).<br>In addition, GDF is actively involved in many international partnerships regarding TB, such as the TB Procurement and Market-Shaping Action Team (TPMAT), where many global health organizations, donors, procurement agents and technical agencies are collaborating in various market-shaping activities.	[11,23,24]
10. No conflict of interest	As explained under B1, the GDF is a third-party organization that operates under the Stop TB Partnership and the UNOPS. The wide variety of stakeholders involved in the Stop TB Partnership, in combination with the fact that GDF operates under the United Nations umbrella provides the GDF the legitimacy and trust to attract funders, buyers, suppliers and technical organizations to participate. Another crucial facilitator is that the GDF and its staff try to maximize the benefits for all its buyers, instead of operating in collaboration with specific buyers or suppliers.	

11.	User-friendliness (both towards buyers and sellers)	According to GDF's annual client satisfaction survey as per ISO Standard Operating Procedures, clients were very satisfied on several areas of GDF's services, scoring 96% satisfaction on the simplicity of GDF's procurement processes, 97% on timely and clear communication), 98% on responsiveness and flexibility of GDF and 99% on the question if they would use GDF's services again.<br>Other factors mentioned under A2, B3, B9, C2 and C3 also contribute to the user-friendliness of GDF's pooled procurement mechanism and related services.	[15,24]
	**C. Suppliers**		
1.	Sufficient number of qualified suppliers	Prior to GDF, there was a lack of quality-assured generic manufacturers for TB medicines. One of the main goals for the GDF to increase access to quality and affordable medicines was market shaping. Based on the market shaping efforts by the GDF, as described under C2, the number of quality-assured suppliers increased from 5 in 2007 to 89 in 2019. However, many factors made it challenging for the GDF to shape a market for (generics) suppliers to enter the TB market:<br>- The market for TB medicines was too small and unpredictable which made it risky and expensive for suppliers to carry stock. Therefore, medicines were often produced to order. Irregular demand (planning) resulted in erratic supply, which in turn resulted in inadequate treatment, and contributed to the emergence of multidrug-resistant TB strains;<br>- The market for TB Medicines was fragmented with many treatment regimens available to treat multidrug-resistant (MDR) TB;<br>- TB treatments have been around since the 1950s, which made it difficult to change and harmonize treatment guidelines;<br>- TB medicines have been mainly produced domestically, which are difficult to assess on quality, and substitute with quality-assured TB medicines, especially because there was no WHO Prequalification program for TB medicines in place.	[15,17,24]
2.	Sufficient production incentives	As part of GDF's market shaping efforts, and to tackle the challenges expressed under C1, GDF has taken multiple approaches to provide sufficient incentives for production of TB medicines, including:<br>- Consolidating demand in low- and middle-income countries and create a market for treatment based on fixed-dose treatments;<br>- Harmonizing treatment guidelines and medicines lists between the WHO, Global Fund and GDF using the TB Medicines Dashboard. These harmonized product lists result in a consolidated market around those specific TB medicines, which in turn incentivize suppliers to produce those;<br>- Establishing a Strategic Rotating Stockpile (SRS) for SLDs to create a buffer and level off the erratic demand of buyers, to share the risk of stock carrying with suppliers and reduce delivery lead times of SLD medicines. In 2020, GDF has started to add FLDs to SRS;	[15,18,29,30,46,47]

	- Securing that TB medicines with relatively low profit margins are exempted from the WHO Prequalification program's annual fees, accounting for 84% of the TB medicines supplied by the GDF. This fee-exemption and the requirements of GDF's quality-assurance policy, described under B8, incentivize suppliers to adhere to either WHO-prequalification or Stringent Regulatory Authority standards if suppliers want to get access to a consolidated TB medicines market.<br>- Providing suppliers long-term framework agreements for 12 months, with option to renew. These framework agreements provide suppliers a certain degree of security to produce, as long as they adhere to the agreed conditions and quality standards;<br>- Financial risk sharing by the GDF through implementing a flexible procurement fund, as explained under B7;<br>- Support innovation and adoption of paediatric-friendly TB medicines, by providing grants to buyer countries, provide technical assistance to change guidelines and carry out accurate demand forecasts.	
3. Sufficient supply incentives	In addition to sufficient production incentives, the GDF has also provided many supply incentives, including:<br>- Providing a predictable, timely and single currency payment mechanism for supplier, as explained in B6;<br>- Providing packaging of TB medicines in 4 languages: English, French Spanish and Russian. This takes the burden of repackaging and translating away from suppliers, making it more attractive to supply these markets;<br>- Taking the burden of distribution from suppliers for MDR-TB, as further explained under C4;<br>- Providing user-friendly services with high client satisfaction ratings, as explained under B11;<br>- The GDF enjoys a positive reputation and has involvement from a wide variety of stakeholders in its operations, as explained under B1 and B9;	[24]
4. Sufficient number of distributors with favourable delivery terms	The GDF has managed to reduce transport costs after consolidating both FLDs and SLDs within one procurement agency (i.e., IDA Foundation). Multiple steps have been taken to reduce freight costs, including:<br>- Increasing the accuracy of demand forecasts. In combination with reduction of lead times and regular deliveries, it reduces emergency orders and air shipments, reducing distribution costs;<br>- Increase delivery from stock instead of ex-supplier deliveries;<br>- Integrate shipments of both FLDs and SLDs to a buyer country;<br><br>In addition, GDF takes the burden of distribution for MDR-TB from suppliers. All MDR-TB suppliers have to do is to ship the orders to the IDA Foundation based in Amsterdam. From there on, GDF/IDA take responsibility for further processing and shipping the order to the buyer country. Once the order is delivered at the agreed point of delivery, the buyer becomes responsible for the order. This responsibility includes import duty, taxes, storage, distribution, and monitoring.	[6,48]

References

1. World Health Organization. Global Tuberculosis Report 2022. Geneva, Switzerland: World Health Organization; 2022. Available from: https://www.who.int/teams/global-tuberculosis-programme/tb-reports/global-tuberculosis-report-2022

2. WHO. What is DOTS? 1999. Report No.: WHO/CDS/CPC/TB/99.270. Available from: https://apps.who.int/iris/bitstream/handle/10665/65979/WHO_CDS_CPC_TB_99.270.pdf;jsessionid=6DC2D51AA628333CE4DD08C3C9642BA1?sequence=1

3. Iseman MD. Tuberculosis therapy: past, present and future. Eur Resp J. 2002;20:87S – 94s.

4. The World Bank. The Stop Tuberculosis Partnership. The World Bank; 2009 Nov. Report No.: 57644. Available from: http://documents.worldbank.org.eur.idm.oclc.org/curated/en/800431468148515795/The-stop-tuberculosis-partnership

5. Raviglione M, Pio A. Evolution of WHO policies for tuberculosis control, 1948–2001. Lancet. 2002;359:775–80.

6. Kumaresan J, Smith I, Arnold V, Evans P. The Global TB Drug Facility: innovative global procurement. Int J Tuberc Lung Dis. 2004;8:130–8.

7. WHO. Report of the Ad Hoc Committee on the Tuberculosis Epidemic. London; 1998 Mar. Report No.: WHO/TB/98.245. Available from: https://apps.who.int/iris/bitstream/handle/10665/63941/WHO_TB_98.245.pdf?sequence=1&isAllowed=y

8. Boseley S. Arata Kochi: shaking up the malaria world. Lancet. 2006;367:1973.

9. World Health Organization. Fifty-third World Health Assembly. Geneva, Switzerland; 2000 May. Report No.: WHA53/2000/REC/3. Available from: https://apps.who.int/iris/bitstream/handle/10665/260193/WHA53-2000-REC3-eng.pdf?sequence=1&isAllowed=y

10. Besada DH, Cooper PAF, Kirton PJJ, Lisk PF. Moving Health Sovereignty in Africa: Disease, Governance, Climate Change. Ashgate Publishing, Ltd.; 2014.

11. Matiru R, Ryan T. The Global Drug Facility: a unique, holistic and pioneering approach to drug procurement and management. Bull World Health Organ. 2007;85:348–53.

12. Management Science for Health. MDS-3: Managing Access to Medicines and Health Technologies (Third Edition). Arlington, VA: Management Science for Health; 2012. Available from: http://apps.who.int/medicinedocs/documents/s19577en/s19577en.pdf

13. United Nations General Assembly. Political declaration of the high-level meeting of the General Assembly on the fight against tuberculosis A/RES/73/3. United Nations; 2018. Available from: https://undocs.org/pdf?symbol=en/A/RES/73/3

14. World Health Organization. Global Tuberculosis Report 2020. Geneva, Switzerland: World Health Organization; 2020. Available from: https://apps.who.int/iris/bitstream/handle/10665/336069/9789240013131-eng.pdf

15. Waning B. GDF Key Updates, Achievements, and Remaining Challenges. Jakarta, Indonesia; 2019. Available from: http://www.stoptb.org/assets/documents/about/cb/meetings/32/32-09%20Global%20Drug%20Facility/32-9.1%20Global%20Drug%20Facility_Presentation.pdf

16. World Health Organization. Tuberculosis: a global emergency. World Health. 1993; Available from: https://apps.who.int/iris/bitstream/handle/10665/52639/WH-1993-Jul-Aug-eng.pdf?sequence=1&isAllowed=y

17. World Health Organization. Global TB drug facility: a global mechanism to ensure uninterrupted access to quality TB drugs for DOTS implementation. World Health Organization; 2001. Report No.: WHO/CDS/STB/2001.10a. Available from: https://apps.who.int/iris/handle/10665/66743

18. Babaley M. Global Drug Facility (GDF) 2020 updates. Virtual Meeting; 2020. Available from: https://extranet.who.int/pqweb/sites/default/files/documents/GDF_2020_updates.pdf

19. US Department of State, Foreign Operations, and Related Programs. Congressional Budget Justification - Fiscal Year 2021. Available from: https://www.usaid.gov/sites/default/files/documents/9276/FY-2021-CBJ-Final.pdf

20. Stop TB Partnership. Stop TB Partnership - Secretariat. Stop TB Partnership. Available from: http://www.stoptb.org/about/secretariat.asp

21. Hauk C, Schäfermann S, Martus P, Muzafarova N, Babaley M, Waning B, et al. Quality assurance in anti-tuberculosis drug procurement by the Stop TB Partnership—Global Drug Facility: Procedures, costs, time requirements, and comparison of assay and dissolution results by manufacturers and by external analysis. PLoS One. 2020;15.

22. Stop TB Partnership. Executive Director's Report n°32. Geneva, Switzerland: Stop TB Partnership; 2019. Report No.: 32. Available from: http://www.stoptb.org/assets/documents/about/cb/meetings/32/32-02%20Report%20of%20the%20Executive%20Director/32-2.1%20Executive%20Director%C2%B4s%20Report.pdf

23. Stop TB Partnership. Stop TB Partnership Annual Report 2014. Geneva, Switzerland: Stop TB Partnership; 2014. Available from: http://www.stoptb.org/assets/documents/resources/publications/annualreports/stoptb_annualeport_2014_web.pdf

24. Boston Consulting Group. TERG Market-Shaping Strategy Mid-Term Review - Final Report. 2019 Jul. Report No.: RFP TGF-19-004. Available from: https://www.theglobalfund.org/media/9235/terg_marketshapingstrategymidterm_review_en.pdf?u=637124310160000000

25. Stop TB Partnership. The Board. Stop TB Partnership. Available from: http://www.stoptb.org/about/cb/

26. Stop TB Partnership. Board Governance Manual. 2023. Available from: https://www.stoptb.org/file/16270/download

27. Blomberg B, Fourie B. Fixed-Dose Combination Drugs for Tuberculosis. Drugs. 2003;63:535–53.

28. Scott C, Gardiner E, de Lucia A. The procurement landscape of pediatric tuberculosis treatment: a Global Drug Facility perspective. Int J Tuberc Lung Dis. 2015;19:S17–22.

29. Stop TB Partnership. Executive Director's Report 2018. Geneva, Switzerland; 2018 Mar. Available from: http://www.stoptb.org/assets/documents/about/cb/meetings/30/30-03%20Report%20of%20the%20Executive%20Director/30-3.1%20Report%20of%20the%20Executive%20Director.pdf

30. IDA Foundation. Invitation to Bid (ITB) for Stop TB/GDF. 2020. Available from: https://www.idafoundation.org/en/blog/post/itb-2020

31. GDF. Buying Quality, Affordable Tuberculosis Drugs through the Global Drug Facility: A Guide to the Direct Procurement Service for Donors, Non-governmental Organizations and Programme Managers. 2007. Available from: http://www.stoptb.org/assets/documents/gdf/whatis/FS%20DP%20Brochure%20FINAL.pdf

32. Global Tuberculosis Programme, World Health Organization. Status of Tuberculosis in the 22 High Burden Countries and Global Constraints to TB Control. 1998. Report No.: WHO/TB/98.242. Available from: https://apps.who.int/iris/bitstream/handle/10665/63872/WHO_TB_98.242.pdf?sequence=1

33. Monsuur V. Pooling procurement of medicines: a comparison of two mechanisms [MSc Thesis]. [Amsterdam, The Netherlands]: VU University Amsterdam; 2020.

34. McKinsey & Company. Evaluation of the Global TB Drug Facility. McKinsey & Company; 2003 Apr. Available from: http://www.stoptb.org/assets/documents/resources/publications/achieve_eval/GDF_Report.pdf

35. Babaley M. GDF, the Global mechanism to facilitate access to affordable quality assured TB medicines and diagnostics, including new tools. 2017. Available from: https://www.usp-pqm.org/sites/default/files/field/image/gdf-m-babaley.pdf

36. GDF. Global Drug Facility Annual Report 2011. Geneva, Switzerland; 2011. Available from: http://www.stoptb.org/assets/documents/gdf/whatis/GDF_Annual_Report_2011_web_lowres.pdf

37. Stop TB Partnership. Global Drug Facility Technical Assistance Update August. Stop TB Partnership. Available from: http://www.stoptb.org/news/stories/2017/ns17_051.asp

38. Waning B. Risks of Decentralized Procurement in Fragile TB Markets: Observations, Implications, and Recommendations at National and Global Levels. The Hague, The Netherlands; 2018.

39. GDF. Quality Assurance Policy. 2010. Available from: http://www.stoptb.org/assets/documents/gdf/drugsupply/GDF%20QA%20Policy%20and%20Procedures.pdf

40. GDF. Budgeting Prices for TB Medicines Q1 2021. 2021. Available from: http://www.stoptb.org/assets/documents/gdf/20210105%20GDF%20TB%20Medicines%20Budgeting%20Prices.pdf

41. GDF. Procurement Request Form for Diagnostics. 2020. Available from: http://stoptb.org/assets/documents/gdf/drugsupply/Procurement%20Request%20Form%20diagnostics%20(English)_Aug%202020.doc

42. GDF. Medicines Catalog. 2020. Available from: http://www.stoptb.org/assets/documents/gdf/drugsupply/GDFMedicinesCatalog.pdf

43. GDF. Adverse Drug Reaction/Serious Adverse Event Form. Available from: http://stoptb.org/assets/documents/gdf/drugsupply/Adverse%20Drug%20Reaction_Serious%20Adverse%20Event%20Form.doc

44. GDF. Procurement and Delivery Planning Guide. 2021. Available from: http://www.stoptb.org/assets/documents/gdf/20210301%20Procurement%20and%20Delivery%20Planning%20Guide.pdf

45. Arinaminpathy N, Cordier-Lassalle T, Lunte K, Dye C. The Global Drug Facility as an intervention in the market for tuberculosis drugs. Bull World Health Organ. 2015;93:237-248A.

46. Stop TB Partnership. The Paradigm Shift 2018-2022. Geneva, Switzerland: Stop TB Partnership; 2019. Available from: http://stoptb.org/assets/documents/global/plan/GPR_2018-2022_Digital.pdf

47. Waning B. New TB Medicines Dashboard. Geneva, Switzerland; 2019. Available from: http://www.stoptb.org/assets/documents/about/cb/meetings/31/31-00%20Welcome/31-0.6%20TB_Meds_Dashboard.pdf

48. GDF. Global Drug Facility Activity Report 2012-2013. Geneva, Switzerland: Stop TB Partnership - WHO; 2014 Oct. Available from: http://www.stoptb.org/assets/documents/resources/publications/annualreports/AR%202012-2013.pdf
