# Supplementary material for: Does structural form matter? A comparative analysis of pooled procurement mechanisms for health commodities
Source: Global Health. 2023 Nov 23;19:90. doi: 10.1186/s12992-023-00974-1 (PMC10668364; doi:10.1186/s12992-023-00974-1)
Supplement: Supplementary file 4 — Additional file 4. [file 12992_2023_974_MOESM4_ESM.pdf]

Early Operational Stage – Asthma Drug Facility (ADF)

General characteristics and historical developments

Asthma, which is a chronic inflammation of the airways, is a non-communicable disease. Although it remains largely unknown what causes asthma, it is believed that a combination of genetic predisposition and environmental factors play a key role [1]. According to a frequently cited study by Masoli et al. [2], there were an estimated 300 million cases of asthma in 2004. That number increased to approximately 339 million in 2017 [3].

In the 1990s, several major studies on asthma prevalence and risk factors were conducted, including the International Study of Asthma and Allergies in Childhood (ISAAC) and The European Community Respiratory Health Survey (EC- RHS), drawing increased global attention to the regional variation in prevalence and the risk factors of asthma [4].

In addition, the International Union Against Tuberculosis and Lung Disease (The Union), which is an international scientific organization, published its first guideline on the management of asthma in adults in 1996. Two years later, The Union conducted a survey, which showed a limited availability of inhalers in 4 of the 8 countries surveyed [5].
Later that same year, experts from six international societies reiterated the need to increase access to effective asthma treatment globally during the first World Asthma Meeting in December 1998 in Barcelona, Spain [4,5]. The lacking affordability and great variability in prices of asthma medicines were further echoed by several surveys of The Union in the early 2000s [5].

Then, in 2001, the Global Drug Facility was established under the Stop TB Partnership, to pooled procure tuberculosis medicines [6]. The executive director of The Union was also involved in setting up the Global Drug Facility. Based on the success of the Global Drug Facility, the executive director of The Union suggested in 2004 to set up a disease-specific pooled procurement mechanism for asthma to increase access to affordable and quality asthma treatment [7].

In the following years, The Union worked towards establishing the Asthma Drug Facility [8]. The ADF was hosted within the Department of Lung Health and Non-Communicable Diseases at the International Union Against Tuberculosis and Lung Disease [9]. The ADF became operational in 2008 [10], and received its first orders from El Salvador and Benin in 2009 [9].

During its early operational years, the ADF had several buyer countries, reaching it highest number of 9 countries in 2012, procuring mainly under pilot projects [11]. However, most of the buyer countries were not procuring with their own funding, but were procuring with external funding (e.g., Global Fund/Practical Approach to Lung Health) or were even granted asthma medicines by the ADF directly [11,12]. This was not a sustainable operation for The Union. Then, in 2013, after a change of leadership at The Union, the Asthma Drug Facility ceased to exist due to a lack of demand and limited financial capacity of buyer countries, according to our respondents.

Essential elements

We have identified several essential elements that have resulted in the discontinuation of the Asthma Drug Facility's pooled procurement mechanism.

1. **The solution the ADF aimed to provide (i.e., quality and affordable essential asthma medicines) was not perceived as a problem by potential buyer countries**

As discussed above, several historical developments have led to the foundation of the Asthma Drug Facility (ADF) by the International Union Against Tuberculosis and Lung Disease (The Union).

The main factors that sparked the idea of setting up the ADF was a combination of the increased recognition in global health arenas of lacking affordable and quality-assured essential asthma treatments in low- and middle-income countries, and the personal experience of The Union's leadership with the Global Drug Facility as a successful pooled procurement mechanism example to increase access to quality and affordable tuberculosis medicines.

Despite this increased global awareness of lacking affordable and quality-assured essential asthma treatments in global health arenas, this problem was not as urgently perceived by potential buyer countries in lower- and middle-income countries.
Multiple reasons have been provided for this lack of perceived urgency of asthma, including a lack of health services around chronic diseases, a lack of access to accurate diagnostics, a lack of accurate demand forecasting of asthma treatments, a lack of context-specific national programs, guidelines and training on asthma, and a lack of knowledge among clinicians and the general public on the diagnosis, disease, treatment and management of asthma [11,13–18].

All these reasons combined resulted in a lack of demand for asthma treatment from buyer countries. As one of our respondents pointed out:

> "Countries did not see what problems Asthma Drug Facility was able to solve for them, because they did not see any problem: for them asthma was not an issue." [Procurement agent 1]

It is important to underline for a pooled procurement mechanism to operate as intended, it is essential that the problem(s) that the pooled procurement mechanism tries to solve need to be explicitly experienced by its buyers.

2. **Limited buyer country budget, both internal and external (through donors)**

Perceiving asthma as a priority problem by buyer countries is particularly important when there is limited funding available in those buyer countries. Often, the limited funding that is available, especially for non-communicable diseases, has to compete with other more urgently perceived diseases.

Another important difference to highlight with the Global Drug Facility's pooled procurement mechanism are the differences in nature of the disease between asthma and tuberculosis (TB). TB, which is a communicable disease, has been on the global health

agenda since the 1970s with the development of the DOTS Strategy, which is integrated approach to tackle TB [19]. The fact that TB can be transmitted from human to human through the air, but curable with the right treatment, has allowed the “TB-sector” to attract a significant amount of external funding from donor organizations and development aid from foreign ministries. On the contrary, asthma, which is a non-communicable disease, has only recently been put on the global agenda, with no cure but only symptom-relief treatment. These characteristics have made it difficult to attract external funding.

For a limited time, some buyer countries (e.g., Guinea Conakry) had the opportunity to procure asthma treatment through the ADF using funds from the Practical Approach to Lung Health (PAL) of their TB grants from the Global Fund to Fight AIDS, TB and Malaria (Global Fund) [17]. However, according to our respondents, the buyer countries lost this opportunity after a financial crisis at the Global Fund, which lead to a policy change on PAL. [Procurement agent 1 & 2]

The ADF tried to secure internal budget in some buyer countries by setting up a revolving drug fund in those countries. Benin, for example, set up a revolving drug fund with the support of the ADF. The idea behind a revolving fund was that it would operate based on selling the asthma treatments to patients in Benin adding a small margin to cover local charges and increase the capital of the revolving fund, so that the fund would become self-financing over time [20]. Although the revolving drug fund contributed to an uninterrupted supply of essential asthma medicines in Benin, several factors have made the replenishment of the revolving drug fund challenging. These factors include the expiry of the procured medicines and administrative inefficiencies (e.g., funding from medicine sales not recovered, medicines provided for free but not recorded, funding from medicine sales recovered but not registered). Also, the extremely underprivileged patients were still unable to buy the asthma inhalers despite the relatively low price, making it difficult to recover the drug fund’s capital investment [20].

3. **Lack of organizational structure with clear roles and responsibilities**

From initiation, the ADF was run by a relatively small in-house team. According to one document, the organizational structure of the ADF consisted of The Union technical team, 2 expert committees (SC and Technical Review Panel) and external technical advisors.

From the beginning, the ADF lacked a dedicated staff. According to several respondents, ADF staff had many other duties, which they tried to combine with their work for the ADF. One respondent mentioned that it was difficult to get work done under these circumstances:

> “So, simple little things like that, you couldn't get resolved, because the ADF was not the priority for the group of staff working on it. They had other things to do.” [Procurement agent 3]

Despite the limited human resources, the ADF did manage to establish a comprehensive qualification process and quality assurance mechanism for asthma treatment, which were not part of the WHO prequalification program. This qualification process was set up to assess the manufacturer’s capacity and inspect their manufacturing site, as well as assess the quality of the products, based on testing the product samples [21].

In addition, according to our respondents, the Asthma Drug Facility, which operated under The Union, had to convince the leadership of The Union every year to continue allocating budget, to keep the ADF operational. After a leadership change at The Union, the new leadership decided to terminate the operations of the ADF because it was too expensive to sustain. Respondents mentioned that this also coincided with closure of the Lung Health and Non-Communicable Diseases department at The Union, shifting The Union's focus from asthma tuberculosis and tobacco-control. [Academic & Procurement agent 2]
This shows that the difficulty to attract buyer countries and external funding made ADF's operations too reliant on The Union, hindering the establishment of an autonomous and sustainable organizational structure at the ADF with clear roles and responsibilities.

4. **Lack of sufficient, predictable and timely budget, both internal (through service fees) and external (through donors)**

The Asthma Drug Facility faced financial shortages to carry out pooled procurement of asthma treatment and to cover organizational expenses.
As mentioned above, buyer countries used mainly three sources to finance their orders through the ADF: external funding (e.g., Global Fund), a revolving drug fund, and ministerial health budgets, which very limited. The combination of a lacking demand and limited financial capacity by buyer countries resulted in The Union to be ADF's main source of financing to carry out procurement. Setting up revolving funds was largely done with capital investments from The Union, and in some other cases, the medicines were provided as grants by The Union under its pilot projects [14,20].

The Union also had to cover ADF's organizational expenses. Initially, the ADF outsourced its procurement services to a procurement agent called the Inter-Agency Procurement Services Office (IAPSO). However, due to the very limited number of incoming orders, the contract with IAPSO was not renewed, because the costs of outsourcing procurement did not outweigh the benefits. After IAPSO, the procurement was done in-house by ADF [8,14]. The ADF secretariat wanted to become self-financing by covering organizational expenses by adding a mark-up to every order. In 2011, the mark-up for ADF's service fee was 10% [21]. However, ADF never reached its goal to become self-financing for the same reason.

The non-communicable nature of the disease and the lack of perceived urgency by low- and middle-income countries made it difficult for the ADF and The Union to attract external funding to carry out procurement or cover organizational expenses.

5. **Supplier incentives**

One of the things that the Asthma Drug Facility was relatively successful doing was to create sufficient incentives for suppliers during its creation and early operational stage to demonstrate willingness to produce and supply buyer countries for a lower price.

Although most of the asthma treatments, including medicines and inhalers, had been off-patent, there was relatively low competition in the generics market for inhalers due to the complexity of the technology. Despite the difficulties of the ADF to aggregate demand, several manufacturers for single ingredient pressurized metered dose inhaler, both originator and generics, have successfully gone through ADF's qualification process [22].

One of the ways the ADF achieved this was by providing sufficient production incentives, or at least its promise. These production incentives mainly incentivized generics suppliers to produce products that were not feasible to invest in before. One of the incentives for generics manufacturers was the possibility to enter new markets that were traditionally dominated by originator companies. In addition, the ADF provided long-term agreements to suppliers that passed the qualification process [21,23], although the duration of these framework agreements remains unknown to us.

Another way for the ADF to incentivize suppliers to enter their qualification process was by providing sufficient supply incentives. These incentives allow suppliers to sell products to markets that have not been feasibly before. This was mainly the case for originator manufacturers. For example, GlaxoSmithKline (GSK), which is the originator manufacturer of Salbutamol pressurized metered dose inhalers, marketed as Ventolin® [17,24], recognized ADF's potential and entered its qualification process to provide asthma inhalers for affordable prices [15,21]. One respondent commented on GSK's participating in ADF's tenders:

> "Right from the beginning actually, they accepted to play the game. … They were the one winning the bid on salbutamol inhalers, at least during the four bids that we set up during the life of ADF, they won four times." [Procurement agent 1]

Later on, our respondent explained what GSK's motive to enter ADF's qualification process might have been:

> "For them it was a tiered pricing policy. They knew the ADF would never be the supplier of the fancy private clinics in the capitals. And they knew there that their business was secure. … It was just their tiered pricing policy for them without mentioning it. And with ADF they were reaching the poorest part of the public sector." [Procurement agent 1]

ADF's efforts resulted in significant price reduction of asthma medicines and inhalers in low- and middle income countries, reaching more than 50% reduction of annual costs per patient with severe asthma in some countries [21,24,25]. However, despite ADF's initial successes in reducing prices and incentivizing manufacturers to produce and supply asthma health products, these incentives were not sufficient. The ADF did not reach a sufficient market size to manage a sustainable pooled procurement mechanism, because of above mentioned reasons such as a lacking demand and limited financial capacity in buyer countries.

Essential elements/actor	Asthma Drug Facility (ADF)	References
**A. Buyers**		
**All buyers <u>need</u> to have individually**:		
1. Perceived problem for which pooled procurement may be a solution (problem)	The Asthma Drug Facility (ADF) has stopped operating, mainly due to a lack of demand from countries. Many reasons have been provided for this lacking demand, including "health services not organised for care of chronic diseases; lack of context-appropriate guidelines based on essential medicines; lack of acceptance by some specialists of essential medicines and the standardised management of asthma; lack of national asthma programmes or training in asthma; the reported influence of big pharmaceutical companies on national procurement and prescribing practices; and national procurement system restrictions on using pooled procurement mechanisms."<br>In addition, one respondent mentioned that: "countries did not see what problems Asthma Drug Facility was able to solve for them, because they did not see any problem: for them asthma was not an issue." [Procurement agent 1]	[13–15,26]
2. Motivations that outweigh the opportunity costs	The motivations to participate did not to outweigh the opportunity costs, because there was a lack of funding and a lack of demand for asthma medicines in the buyer countries. Therefore, ADF had difficulty to incentivize buyer countries to participate in the first place, as expressed one respondent: "So, we [i.e., ADF] were not able to actually convince countries that they should buy these inhalers from the ADF to solve or to improve their programmes. … There was no budget." [Procurement agent 2]	[27]
3. Budget, either internal or external (through donors)	As described under A2, the budget in buyer countries was very limited, because asthma was not a top priority among either global health funding organizations or governments in low- and middle-income countries. In addition, the limited budget that was available for essential asthma medicines had to compete with more expensive asthma medicines that was intended for patients that did not respond to essential asthma medicines.<br>The buyer countries that participated in the ADF's pooled procurement mechanism have mainly used 3 ways of financing their orders:<br>- The Global Fund<br>○ For a limited time, some countries (e.g., Guinea Conakry) could procure medicines through the ADF using funds from the Practical Approach to Lung Health (PAL) of their TB grants from the Global Fund to Fight AIDS, TB and Malaria. However, The Global Fund changed their policy on PAL after a financial crisis at the Global Fund. [Procurement agent 1 & 2]<br>- Revolving Drug Funds<br>○ Other countries, such as Benin, have been supported by the ADF to set up a Revolving Drug Fund, both organizationally and financially. The revolving fund would operate based	[14,17,18,20–22]

	on selling the asthma medicines to patients with adding a small margin to cover local charges and increase the revolving fund, so that the fund would become self-financing in time.<br>○ However, several challenges have been mentioned in Benin regarding the replenishment of the Revolving Drug Fund, such as the expiry of the procured medicines and administrative inefficiencies (e.g., funding from medicine sales not recovered, medicines provided for free but not recorded, funding from medicine sales recovered but not registered). Also, some patients who are extremely poor were still unable to buy the asthma inhalers despite the relatively low price, making it difficult to recover the capital investment.<br>- Health Budgets<br>○ Another way of procuring for buyer countries through the ADF was through health budgets of the Ministry of Health. This type financial commitment was difficult to attract for the ADF, because asthma was not a priority disease in many countries. Only El Salvador committed to procure through ADF using their health budget.	
4. Sufficient technical capacity (e.g., demand forecasting)	Many insufficiencies in technical capacity have been mentioned in low- and middle-income countries that have been part of ADF's pilot project or could be potential buyers. These insufficiencies include a lack of clinical knowledge among health professionals regarding diagnosis, the disease, guidelines and management.<br>Also, potential buyer countries have limited access to accurate diagnostics, making those countries predominantly rely on symptom-based diagnosis. In combination with limited knowledge of health professionals on asthma, symptom-based diagnosis is more susceptible to inaccurate outcomes.<br>One specific example of lacking awareness of health professional in Benin was that some health professionals still prescribe bronchodilators too often, and too few inhaled corticosteroids for patients with persistent asthma. This indicates that not all health professionals are aware of the importance of long-term treatment for asthma patients. In Sudan, challenges were experienced with high turnover of trained staff, resulting in a shortage of trained human resources. Lack of human resources have also been mentioned in Benin.<br><br>Challenges with accurate demand planning have also been recorded. In Benin, some health facilities received fewer asthma patients than planned for, resulting in expiration of inhalers. The mismatch in demand can accumulate to significant wastage of funds, making it more difficult to replenish the Revolving Drug Fund.	[11,13,16–18]

Parmaksiz K, van de Bovenkamp H, Bal R. Does structural form matter? A comparative analysis of four pooled procurement mechanisms. Glob Health. 2023. https://doi.org/10.1186/s12992-023-00974-1.

5. Compatible laws, regulations and policies that allow for (international) pooled procurement	Buyer countries did not always have compatible laws and regulations that allowed for international pooled procurement through ADF. One respondent illustrated the challenges they faced by explaining that ADF performing the tender, agreeing on prices and selecting suppliers was conflicting with national laws, which often state that the Ministry of Health or an organization authorized by the Ministry of Health is responsible for carrying out the procurement. [Procurement agent 1]<br>In addition, Sudan experienced a six-month delay in obtaining customs clearance and official certification for medicines purchased through the ADF, slowing down the implementation of the ADF project in Sudan.	[12,14]
**If buyer's mechanism, all buyers combined, need to have:**		
6. Demonstrated willingness to solve their problem collectively through pooled procurement (shared vision)		
7. Alignment on goals, purpose and operations of the pooled procurement mechanism (shared plan)		
8. Joint need for specific products (product alignment)		
9. Sufficient market size		
10. Sufficient and stable financial capacity		
11. Regulatory harmonization (e.g., shared quality standards, joint assessment, etc.)		
12. Trust (in other buyers and the pooled procurement organization)		
13. Transparent data and information sharing		
14. No history of conflict or failed collaboration		
15. Homogeneity of buyer's characteristics related to their needs		
16. Shared cultural factors and values (e.g., language, traditions, etc.)		
17. Existing political or structural mechanisms		

**B. Pooled procurement organization**		
1. Organizational structure with clear roles and responsibilities	According to one account, the ADF consisted of the Union technical team, 2 expert committees (SC and Technical Review Panel) and external technical advisors. However, the ADF staff did not have clear roles and responsibilities. Several respondents mentioned that there was ADF staff had many other duties, which they tried to combine with their work for the ADF. One respondent mentioned that it was difficult to get work done under these circumstances: "So, simple little things like that, you couldn't get resolved, because the ADF was not the priority for the group of staff working on it. They had other things to do." [Procurement agent 3]<br><br>In addition, the ADF had to convince the International Union Against Tuberculosis and Lung Disease (The Union), under which the ADF operated, to continue allocating budget every year, to keep the ADF operational. When there was a leadership change at The Union, the new board decided to close down the ADF, because it was costing too much money. Respondents mentioned that this also coincided with closure of the Lung Health and Non-Communicable Diseases department at The Union, because The Union decided to focus on tuberculosis and tobacco-control, giving up asthma as a priority. [Academic] & [Procurement agent 2]	[14,27]
2. Clear mandate	The mandate of the Asthma Drug Facility was to provide affordable access to quality- assured essential asthma medicines for low- and middle- income countries. However, the main problem was that this mandate was not provided by the buyer countries, because they did not experience asthma as a priority disease, as expressed under A1. Instead, this mandate was provided by The Union.	[8,20]
3. Standardized and transparent procedures	According to several sources, ADF has set up Standard Operating Procedures for several key operations, including quality-assurance and assessment of manufacturers and products, selection of auditors, pre-shipment inspections, etc. We did not have access to these Standard Operating Procedure documents.<br><br>However, according to several official and unofficial sources, the procurement process of the ADF went as follows:<br>- The ADF health bi-yearly restricted competitive tenders. Only manufacturers with products that had been qualified by the ADF were allowed to enter these tenders;<br>- Manufacturers that were interested in participating in these tenders had to fill in the 'manufacturer questionnaire' and a 'product questionnaire';<br>- These questionnaires needed to be sent to the ADF together with the appropriate certificates and samples of the products the manufacturer wanted to supply;<br>- After assessment and approval by the ADF staff (i.e., qualification process), the suppliers were allowed to enter the tender.	[13,14,16,20,21,26]

	Three pharmacist consultants, which at the time were working for Médecins Sans Frontières according to one respondent [Academic], with experience in quality assurance helped set up the Standard Operating Procedures for quality assessment processes and qualification processes.<br><br>The qualification process of the ADF consisted of two steps:<br>- Assessment of the manufacturer and the manufacturing site<br>o Assessment of the manufacturing site was done by ADF, with ADF bearing the costs for the assessment. The approval was valid for two years.<br>- Assessment of the product based on testing the product samples<br><br>The ADF put great effort into quality assurance, because asthma inhalers were not part of the WHO prequalification program. In addition, many asthma related treatments, such as oral corticosteroids, oxygen, and spacer devices, were not included on the WHO Essential Medicines List.<br><br>Regarding in-country product registration, the ADF operated solely on pilot level. During these pilots, the ADF mainly provided medicines as grants. Therefore, the orders could be imported into the countries using waivers, without registering the product.	
4. Sufficient, predictable and timely budget, either internal (through service fees) or external (through donors) to carry out pooled procurement	The ADF was mainly funded by the International Union Against Tuberculosis and Lung Disease (The Union). In turn, The Union has been funded by several Ministries of Health, National Lung foundations, and other non-governmental organizations.<br>However, the funding was very limited. It had difficulty attracting funding from international donors, governments and global health organizations. This was partially due to a lack of demand in recipient countries and also due to the nature of the disease. Asthma, as a non-communicable disease, was given lower priority compared to respiratory diseases of communicable nature, such as tuberculosis.<br><br>In addition, as described under A3, there were mainly three ways that buyer countries participated in the ADF's pooled procurement mechanism: external funding (e.g., The Global Fund), revolving drug fund and health budgets from buyer countries, which were very limited. The main source of financing came from The Union itself. Setting up these revolving drug funds was mainly done with capital investment from The Union, and in other cases the medicines were provided as grants by The Union/ADF.	[14,16,20,26]

5.	Sufficient, predictable and timely budget, either internal (through service fees) or external (through donors), to cover organizational expenses	The ADF was managed by the Department of Lung Health and Non-Communicable Diseases and funded by The Union. As mentioned under A4, The Union had difficulty attracting financial support from other organization for the ADF, resulting in The Union bearing the costs of ADF's operations, essentially.<br><br>The goal for the ADF Secretariat was to become self-financing with adding a surcharge on every order. In 2011, this surcharge for ADF's service fees was 10%. However, due to the limited orders, ADF did never reach this goal of becoming self-financing. In fact, as described under A1, the ADF had to convince The Union to continue allocating budget every year.	[9,14,21,26]
6.	Predictable, timely and efficient payment mechanism	As explained under A3, buyers have mainly used three ways of financing their orders through ADF: external funding (e.g., The Global Fund) revolving drug fund and health budgets from buyer countries.<br>Although we found little information on the actual payment mechanism of the ADF, some documents mentioned that buyers had to make a full payment to ADF in advance of the order, by bank transfer or a letter of credit from a bank.	[26,28]
7.	Human resources (sufficient in numbers and expertise)	As mentioned under B1, the ADF staff had other responsibilities, which they tried to combine with their work for the ADF. One respondent mentioned: "It was run with really very small human resources. There was a lot of work done by a very few number of people." [Academic]	[14]
8.	Sufficient technical capacity (e.g., procurement, quality assessment, forecasting, etc.)	At initiation, ADF outsourced all steps of the procurement process to the Inter-Agency Procurement Services Office (IAPSO). Due to the very limited number of incoming orders, the contract with IAPSO was not renewed because the costs of outsourcing to a procurement agent did not outweigh the benefits it provided. After IAPSO, the procurement was done in-house by ADF.<br>ADF also tried to provide additional services to buying countries. The Union offered a combination of services to improve the management of asthma in health services, containing training materials, an information system and a technical guide to improve quality services.	[5,14,16,21,27]
9.	Positive reputation	Although ADF did achieve positive outcomes in terms of lower priced and quality-assured asthma products, it did not achieve financial sustainability to operate long enough to build a positive reputation. This was also due to a lack of demand of buyer countries, as described under A1.	[24]
10.	No conflict of interest	We found little information on potential conflict of interest. Although the ADF staff had to balance their work for ADF with other duties at The Union, we do not have any indications that this compromised the independence of the staff or led to any conflict of interest.	
11.	User-friendliness (both towards buyers and sellers)	Although the ADF tried to provide affordable and quality-assured asthma medicines, in combination with a more rounded procurement service as described under B8, procuring medicines was conditional, including:<br>- The buyer countries were responsible of importing the medicine;<br>- Medicines were only allowed to be sold by adding a minimal mark-up<br>- Medicines were not allowed to be export or resold	[28]

	- As mentioned under B6, the buyer had to pay ADF in advance<br>- Submit routine monitoring reports to the ADF. These monitoring reports had to include the classification that were used for symptoms and for patients treated with corticosteroids procured through ADF, buyers had to provide data collected during initial visit and annual follow-up visits<br>Although we found little data, we expect that some buying countries lacking sufficient technical capacity, infrastructure and human resources, might have had difficulties to adhere to these conditions, discouraging them to procure through ADF using their own budget.<br><br>We found little information on ADF's user-friendliness towards suppliers. The Standard Operating Procedures described under B3 might have facilitated a transparent and predictable process, but the comprehensive qualification process might also have discouraged suppliers to participate in tenders.	
**C. Suppliers**		
1. Sufficient number of qualified suppliers	Although asthma medicines and inhalers have been off-patent, generic manufacturers have had difficulty developing quality inhalers and turbo inhalers due to its complex nature. Challenges were experienced with defining the right combination of diameters of the metering valve and the actuator nozzle to dispense the right size of fine particles. Therefore, not many generic manufacturers have been producing these products. One of the primary goals of the ADF was to aggregate demand and create a market and incentivize generic manufacturers to invest and produce these asthma products, as explained under C2.<br>Despite the difficulty of developing quality inhalers for generic manufacturers, as mentioned above, several innovator and generic manufacturers have entered ADF's qualification process during its years of operation. The manufacturers for single ingredient pressurized metered dose inhaler (pMDIs) that have successfully gone through ADF's qualification process were based in the United Kingdom (i.e., GlaxoSmithKline), Spain, India (i.e., Cipla), and Bangladesh (i.e., Beximco Pharmaceuticals Ltd).	[15–17,22,23,25,27,29]

2.	Sufficient production incentives	ADF aimed to incentivize generic manufacturers to invest and produce asthma inhalers by aggregate demand in lower- and middle-income countries, and create a market for asthma medicines and inhalers that was practically non-existing. Generic manufacturers were incentivized because there was a possibility for entry to new markets, where traditionally innovator companies dominated.<br>In addition, the Asthma Drug Facility provided long-term agreements to suppliers that passed the qualification process, although the duration of these framework agreements remains unknown to us. Long-term agreements incentivize suppliers to invest in producing certain products, because they provide suppliers the security that their products will be procured, given that these products remain to meet the quality criteria.<br>Although ADF initially incentivized manufacturers to produce asthma health products and participate in tenders, these production incentives were not sufficient. The ADF never reached a sufficient market size to manage sustainable pooled procurement mechanism, because demand was lacking in buyer countries and buyers lacked financial capacity to procure asthma medicines, as explained under A1-A3.	[14,16,21,23]
3.	Sufficient supply incentives	The Asthma Drug Facility also tried to incentivize innovator companies to supply their products to for an affordable price to new markets in lower- and middle-income countries.<br>For example, GlaxoSmithKline (GSK), which was an originator manufacturer, recognized ADF's potential, and therefore might have been willing to participate in ADF's tenders and lower their prices. These manufacturers wanted to be involved in case ADF would expand and develop into a pooled procurement mechanism which access to low- and middle-income markets.<br>One respondent commented on GSK's participating in ADF's tenders: "Right from the beginning actually, they accepted to play the game. … They were the one winning the bid on salbutamol inhalers, at least during the four bids that we set up during the life of ADF, they won four times." [Procurement agent 1]	[14]
4.	Sufficient number of distributors with favourable delivery terms	Delivery was initially organized by ADF's procurement agent, IAPSO. We have no information how the delivery was organized after the expiration of the contract with IAPSO.<br>The final price of medicine procured through the ADF differed per country due to differences distribution costs, pre-shipment inspection, import taxes, custom clearance costs and other costs depending on local factors. For example, in Benin, a small margin was agreed to be added on the medicine price to cover the costs of storage, distribution, custom clearance, potential loss of drugs and to replenish and grow to the revolving drug fund.	[5,8,20,21,21,27,30]

References

1. Braman SS. The Global Burden of Asthma. Chest. 2006;130:4S-12S.

2. Masoli M, Fabian D, Holt S, Beasley R. The global burden of asthma: executive summary of the GINA Dissemination Committee Report. Allergy. 2004;59:469–78.

3. Vos T, Abajobir AA, Abate KH, Abbafati C, Abbas KM, Abd-Allah F, et al. Global, regional, and national incidence, prevalence, and years lived with disability for 328 diseases and injuries for 195 countries, 1990–2016: a systematic analysis for the Global Burden of Disease Study 2016. Lancet. 2017;390:1211–59.

4. Sterk PJ, Buist SA, Woolcock AJ, Marks GB, Platts-Mills TA, Mutius E von, et al. The message from the World Asthma Meeting. The Working Groups of the World Asthma Meeting, held in Barcelona, Spain, December 9-13, 1998. European Respiratory Journal. 1999;14:1435–53.

5. Ait-Khaled N, Enarson DA, Bissell K, Billo NE. Access to inhaled corticosteroids is key to improving quality of care for asthma in developing countries. Allergy. 2007;62:230–6.

6. Kumaresan J, Smith I, Arnold V, Evans P. The Global TB Drug Facility: innovative global procurement. Int J Tuberc Lung Dis. 2004;8:130–8.

7. Billo NE. Do we need an Asthma Drug Facility? Int J Tuberc Lung Dis. 2004;8:391–391.

8. Billo NE. Asthma Drug Facility: from concept to reality. Int J Tuberc Lung Dis. 2006;10:709–709.

9. The Union. Activity Report 2009. International Union Against Tuberculosis and Lung Disease; 2009.

10. The Union. Activity Report 2008. International Union Against Tuberculosis and Lung Disease; 2008.

11. The Union. Activity Report 2012. International Union Against Tuberculosis and Lung Disease; 2012.

12. The Union. Activity Report 2011. International Union Against Tuberculosis and Lung Disease; 2011.

13. Bissell K, Perrin C, Beran D. Access to essential medicines to treat chronic respiratory disease in low-income countries. Int J Tuberc Lung Dis. 2016;20:717–28.

14. Monsuur V. Pooling procurement of medicines: a comparison of two mechanisms [MSc Thesis]. [Amsterdam, The Netherlands]: VU University Amsterdam; 2020.

15. Perrin C. Access to Essential Medicines for Asthma. Presentation at: WHO Technical Briefing Seminar on Essential Medicines & Health Products. 2013. Available from: https://slideplayer.com/slide/4549199/

16. Beran D, Zar HJ, Perrin C, Menezes AM, Burney P. Burden of asthma and chronic obstructive pulmonary disease and access to essential medicines in low-income and middle-income countries. Lancet Respir Med. 2015;3:159–70.

17. International Union against Tuberculosis and Lung Disease. The global asthma report 2011. Paris, France: International Union Against Tuberculosis and Lung Disease; 2011. Available from: http://www.globalasthmareport.org/sites/default/files/Global_Asthma_Report_2011.pdf

18. The Global Asthma Network. Global Asthma Report 2018. Auckland, New Zealand: The Global Asthma Network; 2018. Available from: https://theunion.org/sites/default/files/2020-08/Global%20Asthma%20Report%202018.pdf

19. WHO. What is DOTS? 1999. Report No.: WHO/CDS/CPC/TB/99.270. Available from: https://apps.who.int/iris/bitstream/handle/10665/65979/WHO_CDS_CPC_TB_99.270.pdf;jsessionid=6DC2D51AA628333CE4DD08C3C9642BA1?sequence=1

20. Agodokpessi G, Aït-Khaled N, Gninafon M, Tawo L, Bekou W, Perrin C, et al. Assessment of a revolving drug fund for essential asthma medicines in Benin. J Pharm Policy Pract. 2015;8. Available from: https://www.ncbi.nlm.nih.gov/pmc/articles/PMC4394562/

21. Macé C, Bissell K, Billo NE. Access to essential asthma medicines: the response of the Asthma Drug Facility. Essential Medicines Monitor. 2011;

22. The Global Asthma Network. Global Asthma Report 2014. Auckland, New Zealand: The Global Asthma Network; 2014. Available from: http://www.globalasthmareport.org/resources/Global_Asthma_Report_2014.pdf

23. Beximco Pharmaceuticals LTD. Annual Report 2008. Beximco Pharmaceuticals LTD; 2009. Available from: https://beximcopharma.com/images/stories/investor/pdf/ARP-2008.pdf

24. Babar Z-U-D, Lessing C, Mace C, Bissell K. The availability, pricing and affordability of three essential asthma medicines in 52 low- and middle-income countries. Pharmacoeconomics. 2013;31:1063–82.

25. Ramakant B. Access to medicines is key to reducing suffering from asthma. Citizen News Service - CNS. 2013 May 7; Available from: https://www.citizen-news.org/2013/05/access-to-medicines-is-key-to-reducing.html

26. Chiang C-Y, Bissell K, Macé C, Perrin C, Marks G, Mortimer K, et al. The Asthma Drug Facility and the future management of asthma. Int J Tuberc Lung Dis. 2022;26:388–91.

27. Billo NE. Asthma Drug Facility. Presentation at: GARD Meeting. Seoul, South Korea; 2007.

28. Chiang C-Y. Improving asthma management in the Asia-Pacific region. [Presentation]. Hong Kong; 2011.

29. Virchow JC, Akdis CA, Darba J, Dekhuijzen R, Hartl S, Kobelt G, et al. A review of the value of innovation in inhalers for COPD and asthma. J Mark Access Health Policy. 2015;3.

30. Billo NE. Good news: asthma medicines for all. Int J Tuberc Lung Dis. 2010;14:524–524.
